# Quantitative Extraction and Evaluation of Tomato Fruit Phenotypes Based on Image Recognition

**DOI:** 10.3389/fpls.2022.859290

**Published:** 2022-04-13

**Authors:** Yihang Zhu, Qing Gu, Yiying Zhao, Hongjian Wan, Rongqing Wang, Xiaobin Zhang, Yuan Cheng

**Affiliations:** ^1^Institute of Digital Agriculture, Zhejiang Academy of Agricultural Sciences, Hangzhou, China; ^2^State Key Laboratory for Quality and Safety of Agro-Products, Institute of Vegetables, Zhejiang Academy of Agricultural Sciences, Hangzhou, China

**Keywords:** quantitative, tomato fruit, phenotyping, image recognition, deep learning

## Abstract

Tomato fruit phenotypes are important agronomic traits in tomato breeding as a reference index. The traditional measurement methods based on manual observation, however, limit the high-throughput data collection of tomato fruit morphologies. In this study, fruits of 10 different tomato cultivars with considerable differences in fruit color, size, and other morphological characters were selected as samples. Constant illumination condition was applied to take images of the selected tomato fruit samples. Based on image recognition, automated methods for measuring color and size indicators of tomato fruit phenotypes were proposed. A deep learning model based on Mask Region-Convolutional Neural Network (R-CNN) was trained and tested to analyze the internal structure indicators of tomato fruit. The results revealed that the combined use of these methods can extract various important fruit phenotypes of tomato, including fruit color, horizontal and vertical diameters, top and navel angles, locule number, and pericarp thickness, automatically. Considering several corrections of missing and wrong segmentation cases in practice, the average precision of the deep learning model is more than 0.95 in practice. This suggests a promising locule segmentation and counting performance. Vertical/horizontal ratio (fruit shape index) and locule area proportion were also calculated based on the data collected here. The measurement precision was comparable to manual operation, and the measurement efficiency was highly improved. The results of this study will provide a new option for more accurate and efficient tomato fruit phenotyping, which can effectively avoid artificial error and increase the support efficiency of relevant data in the future breeding work of tomato and other fruit crops.

## Introduction

Tomato (*Solanum lycopersicum* L.) is one of the most widely consumed vegetables around the world (Kaya et al., 2020; Alam et al., 2021; Faizan et al., 2021). Tomatoes are used for food in a variety of forms and contain considerable vitamins A, C, and lycopene, which have been shown to reduce the risk of cancer and neurodegenerative disorder (Kinkade and Foolad, 2013; Caseiro et al., 2020). Due to the genetic diversity and commercial value, tomato is also a model species for fruit development studies. Since tomato spread out through trade from South America and Mesoamerica where tomato domestication began, it was chosen for diverse fruit colors and sizes throughout breeding. Consequently, current tomato cultivars have various phenotypes including colors, sizes, and internal structure (Sierra-Orozco et al., 2021). For example, cultivated tomatoes have colors from green to orange, red, and black; sizes from 6 to 100 mm, and even larger; and internal shapes including diverse locule and pericarp traits. All these traits determine the market valuation and culinary consumption procedures of tomato fruits, including fresh, sliced, diced, or cooked (De Corato, 2020).

The growers’ demand for lucrative tomatoes is associated with the color and size of the tomato fruits. Although all traits are crucial, consumers prefer to rate tomatoes initially on their sensory appearance and then on their flavor. For example, large-sized and flat tomatoes are preferred to be sliced or cooked because they are easier for manual processing. Thus, fruit size and vertical/horizontal ratio (fruit shape index) are important indicators of tomato traits. Fruit color is the most attractive attribute as commodities (Oltman et al., 2014). The pericarp is the circular outer part of the fruit fresh part developed from mesocarp. Locules are septum-enclosed independent spaces separated by the placenta which are filled with semiliquid tissue. Pericarp and locules determine the taste and malformation rate. Fruits with large locule area proportion tend to have higher moisture content and are suitable for fresh-eating, whereas fruits with small locules and thick pericarp are preferred for cooking (Tamasi et al., 2019). Because tomato fruit traits are so important to customers, tomato breeders are working hard to improve those qualities. A deeper knowledge of tomato fruit phenotypes can help in breeding attempts to enhance fruit quality.

Although the tomato genome has been sequenced for many years (The Tomato Genome Consortium, 2012) and many quantitative trait loci (QTLs) related to fruit morphology have been identified (Prudent et al., 2009; Celik et al., 2017), traditional breeding techniques are still dominant in tomato breeding, and the traits of parents for hybrid breeding are selected mainly depending on breeder’s experience (Zhu et al., 2018). Such multidimensional screening of tomato phenotypes is not only time-consuming and labor-intensive but also limits the breeding accuracy and efficiency. Therefore, it is necessary to develop methods to quantitatively evaluate the tomato fruit phenotypes, including the fruit color, size, morphology, and locule structures (Bhatta et al., 2021). Moreover, as the concept of breeding 4.0 being raised, which seeks out the desirable traits combined with the aid of artificial intelligence based on fully comprehended bioinformatic and agronomic data (Wallace et al., 2018), high-throughput and quantitative phenotyping is more likely to promote crop breeding efficiency in a novel, data-driven way (Washburn et al., 2019).

Tomato fruit traits pertaining to morphological and structural aspects are used for fruit phenotyping (Darrigues et al., 2008), which is closely connected with genetic diversity analysis regarding the effects of breeding and crop genetic resources conservation and exploitation (Mata-Nicolás et al., 2020). With the progress of image recognition based on deep learning methods, quantitative and high-throughput plant phenotyping is becoming promising (Pereira et al., 2021). There are plenty of successful studies in most field crops (Li et al., 2020), but using high-throughput methods to assess quantitative phenotypes of vegetables is still at an earlier experimental stage (Tripodi et al., 2018; Boogaard et al., 2020). Most vegetable breeding initiatives appear to be hampered by a lack of affordable and accessible high-throughput techniques. Generally, manual fruit measurement, which is commonly utilized in traditional phenotypes evaluation and costs a lot of labor and time, is the main barrier in comprehensive tomato fruit phenotyping (Costa et al., 2018). A tool called *Tomato Analyzer* based on computer vision has shown to be quite effective in extracting tomato fruit morphometric and structural features automatically, yet the analysis of some fruit internal structures, such as locule number and area, still requires manual operations (Gonzalo et al., 2009). Another tool called *LocAnalyzer* attempts to count tomato fruit locule automatically with the help of computer vision recognition (Spetale et al., 2020), but it only processes one fruit in each image and requires image scanning of cut fruits.

In this study, a series of quantitative indicators (color, diameters, angles, locules, and pericarp) for tomato fruit phenotypes are proposed based on the quantitative requirements of tomato breeding. A combination of image recognition and Mask Region-Convolutional Neural Network (R-CNN) deep learning methods to extract these indicators is proposed. The findings are supposed to provide a useful tool for the high-throughput phenotyping of tomatoes. The tool also assists breeders to evaluate tomato fruit traits quantitatively and automatically and could be extended to other fruit crop breeding.

## Materials and Methods

### Materials, Sampling, and Image Acquisition

**FIGURE 1:**
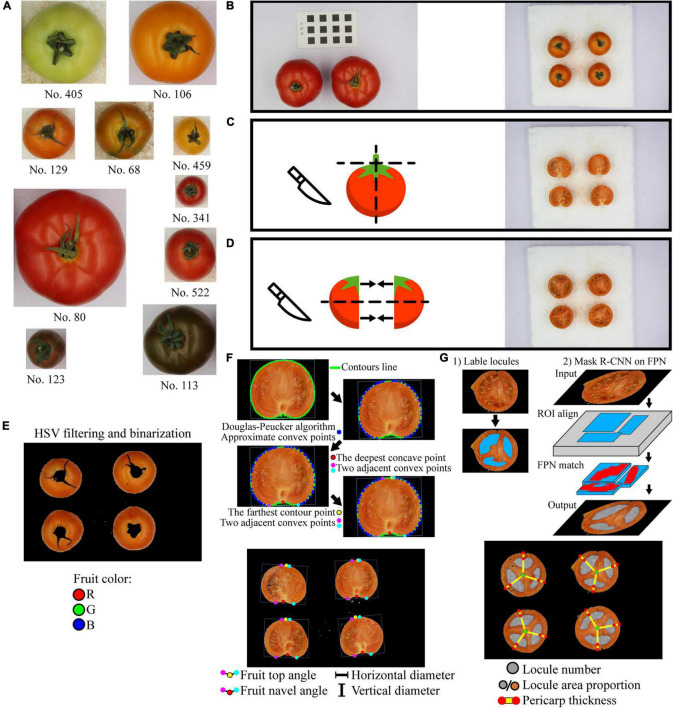
Tomato samples and image acquisition process. **(A)** Tomato samples, images are proportional; **(B)** Image acquisition of ruler card and the intact fruit; **(C)** Image acquisition of the vertical cut; **(D)** Image acquisition of the horizontal cut; the same fruit of vertical cut is cut then joint the corresponding horizontal parts together. **(E)** The extraction of the fruit average color Red, Green, and Blue (R, G, B) values from the intact fruit images; **(F)** Fruit top angle, fruit navel angle, horizontal diameter, and vertical diameter extraction from the vertical cut images; the red, yellow, magenta, and cyan points are the navel point, the top point, the left, and the right adjacent convex points, respectively; **(G)** Fruit locule number, locule area proportion, and pericarp thickness extraction from the horizontal cut images; the gray and orange circles refer to the locule segmentation and the horizontal cut section, the green points are the center of the horizontal cut section, and the red points are the intersections of the lines and the edges.

The ripe fruits of all 591 tomato cultivars were applied for image acquisition and phenotyping. Ten cultivars with obvious differences in fruit size, fruit color, and other aspects were selected as samples for phenotyping comparison and verification (Table 1 and Figure 1A). All tomato cultivars were cultivated in the tomato germplasm field of Zhejiang Academy of Agricultural Sciences, Yangdu, Haining City, Zhejiang, China (120.411°N, 30.441°E).

**TABLE 1:** Tomato samples for image acquisition and phenotyping verification.

Cultivar no.	Fruit color^a^	First harvest/d^b^	Botanical name
No. 405	Green	112.8	*Solanum lycopersicum* L.
No. 459	Yellow	103.2	*Solanum lycopersicum* L. var. cerasiforme (Alef.) Voss
No. 106	Orange	118.0	*Solanum* spp.
No. 68	Orange	129.0	*Solanum lycopersicum* L.
No. 129	Orange	116.3	*Solanum lycopersicum* L.
No. 522	Red	111.2	*Solanum lycopersicum* L.
No. 80	Red	107.6	*Solanum lycopersicum* L.
No. 341	Red	126.2	*Solanum lycopersicum* L.
No. 123	Black	105.6	*Solanum* spp.
No. 113	Black	115.0	*Solanum* spp.

*^a^Fruit color is based on human eye sensory instead of accurate color types.*

*^b^The average number of days between planting and first harvest.*

Three to four representative tomato fruit samples were placed on a white background at intervals. A black-white ruler card with accurate squares of 1 cm × 1 cm was attached next to the samples as reference (Figure 1B). Images of both the samples and the ruler card were acquired using a camera (Canon EOS 20D, 50 mm fixed-focus lens, Canon, Inc., Tokyo, Japan) in a photo box with a diffuse inner surface and a soft light (24 W, color temperature 6,000 K, WenaSelin Tec., Co. Ltd., Hangzhou, China) on the top. Due to the fixed focal length, the image with the ruler card was taken only once to calibrate the measurement before taking other images. After taking an image of the intact tomato fruits (Figure 1B), each fruit was sepal-removed and cut vertically through the center. Then, another image of the vertical cut was taken (Figure 1C). Finally, both the vertical halves were cut horizontally, and the adjacent parts were put together to take a third image of the horizontal cut (Figure 1D).

### Image Recognition and Phenotyping Indicators

To quantitatively extract tomato fruit phenotyping indicators from the images, OpenCV 4.5.3 computer vision library was applied to conduct image recognition tasks including background filtering, length measuring, and contour points locating. The tomato fruit locules were recognized using a deep learning instance segmentation model developed on PaddlePaddle 1.8.4 (Ma et al., 2019).

#### Intact Tomato Fruits

The image recognition process for the image of intact tomato fruits is shown in Figure 1E. The image was first transformed into Hue-Saturation-Value (HSV) color mode. Based on the saturation and value differences between the tomato fruits and the background, HSV filtering and binarization were then performed to remove the background. Pixels that had H between 0 and 180° (ranging 0–360°) and both S (ranging 0–255°) and V (ranging 0–255°) greater than a certain threshold were marked as tomato fruit area, and other pixels were background. According to our preliminary experiment, such a threshold would vary among cultivars. Still, for a certain fruit color type, the threshold stays the same. As background pixels were replaced with black color, the image was binarized, and only fruits were remained. Then, the fruit color in Red, Green, and Blue (RGB) color mode was extracted for each fruit in the image and taken the average R, G, and B value, respectively.

#### Vertical Cut

The image recognition process for the image of vertical cut is shown in Figure 1F. HSV filtering and binarization were performed first, as mentioned above. The fruit contours in the image and the corresponding minimum area rectangles enclosing the fruit contours were extracted. According to our preliminary experiment, the fruit horizontal diameters extracted from intact fruit and vertical cut image have no significant difference. Thus, the width and height of the rectangle were measured and recorded as the tomato fruit horizontal diameter and vertical diameter, respectively. Vertical/horizontal ratio (also called fruit shape index) is calculated as vertical diameter divided by horizontal diameter. Fruit navel angle and fruit top angle are the angles of the calyx indentation and top protrusion on the vertical section, respectively. They were measured as follows:

1. Find the fruit contour;
2. Use the Douglas-Peucker algorithm (Douglas and Peucker, 1973) to locate the approximate convex points;
3. Find the concave point on the contour that had the largest distance toward the line passing through its two adjacent convex points. This concave point is marked as the fruit navel point (red points in Figure 1F);
4. In rare cases, the abovementioned concave point is not the real fruit navel point. Crop the image region between each concave point and the center point of fruit contour. Under HSV color mode, find the concave point that has the lowest saturation in the cropped region. This concave point is the fruit navel point because the region near calyx on the vertical section is whiter than other fruit parts;
5. Find the farthest contour point on the opposite side of the contour. This is the fruit top point;
6. Find two adjacent convex points of both the fruit navel point and the fruit top point. Calculate the fruit navel angle and fruit top angle which are denoted in Figure 1F;
7. The top/navel ratio is calculated as fruit top angle divided by fruit navel angle.

#### Horizontal Cut

The image recognition process for the image of horizontal cut is shown in Figure 1G. The locules in tomato fruits were segmented using a deep learning instance segmentation model based on Mask R-CNN (He et al., 2018). The procedures are as follows.

##### Locules Labeling

Among all cultivars, 335 images of horizontal cut tomato fruits were selected to form a fruit locule image dataset. All locules in the images were manually labeled as polygons using an image annotation tool LabelMe (Russell et al., 2008).

##### Data Augmentation

Increasing the number of training images by transformation and enhancement techniques is useful to avoid the overfitting and non-convergence of R-CNN algorithms. Images rotating 90, 180, and 270° were appended into the dataset. Vertical and horizontal mirroring of images was also appended into the dataset. Images were resized into 0.1, 0.25, 0.5, 0.8, 2, and 4 times of the raw image size with bilinear interpolation. These images were appended into the dataset to augment image size levels. Besides the above transformation techniques, image blurry was applied as an enhancement technique to improve the model performance on blurred images. All the above mentioned transformed images were processed by Gaussian blurry with 5 × 5 kernel and appended into the dataset. The number of the image dataset was increased to 8,040 after the abovementioned augmentation methods. The dataset was then divided into training (5,628 images, 70%), validation (1,608 images, 20%), and testing (804 images, 10%) sets.

##### Deep Learning Model Training

Mask R-CNN has been wildly applied in fruit detecting (Gao et al., 2020) and segmentation (Liu et al., 2020), but the network model for tomato fruit locule segmentation still needs further training to improve the performance. Feature Pyramid Network (FPN) was implemented to fuse color and texture features into the model (Lin et al., 2017). Region Proposal Network (RPN) was applied to generate region proposals based on the features, which were aligned with the images through the Region of Interest (RoI) Align process (Tang et al., 2018). The model was then constructed by feeding the RoI Aligned features to the convolution layers for segmentation. During training, a positive segmentation was confirmed if Intersection over Union (IoU) is greater than 0.5. The loss function was defined as the sum of the bounding box regression loss. The termination condition is when the loss function value remained consistent or it hit 5,000 iterations.

##### Deep Learning Model Evaluation

To evaluate the segmentation results, all segmentation results were divided into four types, namely, true positive (TP), true negative (TN), false positive (FP, wrong segmentation), and false negative (FN, missing segmentation). Precision (***P***), recall (***R***), and *F*1*-score* (the harmonic average of ***P*** and ***R***) were defined as follows:


$$\text{P}=\frac{\text{T}\,P}{\text{T}\,P+\text{F}\,P}$$

$$\text{R}=\frac{\text{T}\,P}{\text{T}\,P+\text{F}\,N}$$

$$\text{F1}-\text{s}\,c\,o\,r\,e=\frac{2\times \text{P}\times \text{R}}{\text{P}+\text{R}}$$

Since there was only one segmentation class, average precision (***AP***) was defined as the area under the curve plotted with the ***P*** at the vertical axis and the ***R*** at the horizontal axis.

##### Phenotyping Indicators Extraction

The locule number was consequently the number of locule segmentations after the deep learning model recognition. The locule area proportion was the area proportion of all locule segmentations in one fruit over the corresponding fruit horizontal cut section. For each fruit, a line was drawn through the center of the horizontal cut section and each center of the locule segmentation. Each line intersects the edge of each locule segmentation and the horizontal cut section at a pair of points (red points in Figure 1F). Pericarp thickness was thus measured as the average of the lengths between these pairs of points.

### Verification

Among all the phenotyping indicators, the tomato fruit horizontal diameter, vertical diameter, pericarp thickness, and locule number could be obtained by direct measurement. Thus, these indicators were manually counted or measured to make a comparison with the results obtained by image recognition. Unequal variance tests (Levene’s test) and significance tests were conducted between the results of the manual measurement and the image recognition. The Root Mean Square Error (***RMSE***) was applied to evaluate the accuracy of the image recognition as follows:


$$\text{R}\,M\,S\,E=\sqrt{\frac{1}{\text{n}}\,\sum_{i=1}^{n}{\left(\frac{{\text{R}}_{i}-{\text{M}}_{i}}{{\text{M}}_{i}}\right)}^{2}}$$

where *n* represents the number of measurements, *R*_*i*_ represents the image recognition result, and *M*_*i*_ represents the manual result. All statistical calculations and analyses were conducted using the R programming language (version 4.0.5) (R Core Team, 2020). The C# code for the methods is presented in Supplementary Material “used_codes.cs.”

## Results

### Tomato Fruit Color

**FIGURE 2:**
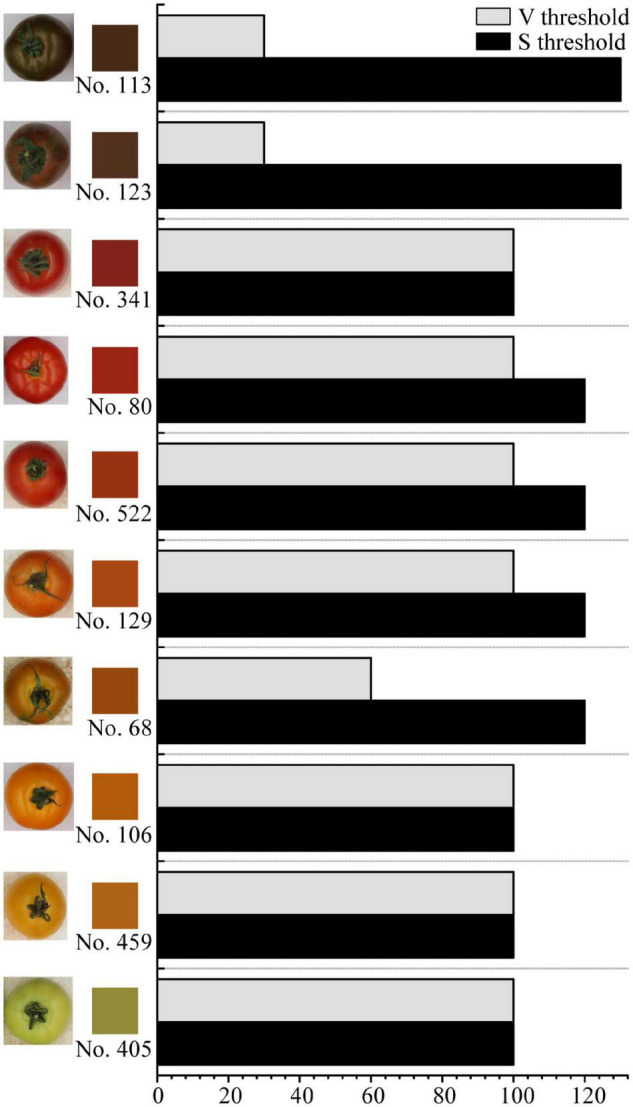
Fruit average colors and thresholds for Hue-Saturation-Value (HSV) filtering and binarization of the intact fruit images. The square beside each tomato fruit image shows the fruit average color in RGB mode. Fruit images are not proportional.

The threshold for HSV filtering and binarization is crucial to tomato fruit color recognition. For green, orange, red, and black tomato fruits, as sorted in colors in Figure 2, the H thresholds for HSV filtering keep the same while S and V thresholds change in a range of 30–130. However, the S threshold varies in a minimal range from 100 to 120, and the V threshold stays the same at 100 for most samples, except that the black tomatoes (No. 113, No. 123) need S and V thresholds of 30 and 130, and No. 68, which has mottle on the surface, needs S thresholds of 60. This suggests that there are certain S and V threshold combinations for the HSV filtering and binarization of pure color and black tomatoes. Since No. 68 is the only mottled tomatoes found among all cultivars, the S and V threshold combination (120, 60) should be considered as a simple case. Notably, the sepal and calyx are also marked as background and removed, and this is designed in the HSV filtering and binarization process so that the sepal and calyx do not influence the extraction of the fruit color R, G, and B values. As is shown in Figure 2, the fruit average color is objective and in accordance with the human eye sensory observation, suggesting that the fruit color obtained by image recognition is reliable and not subjected to human judgment. The detailed fruit color R, G, and B values are listed in Supplementary Table 1.

### Performance of Phenotyping From Vertical Cut Images

**FIGURE 3:**
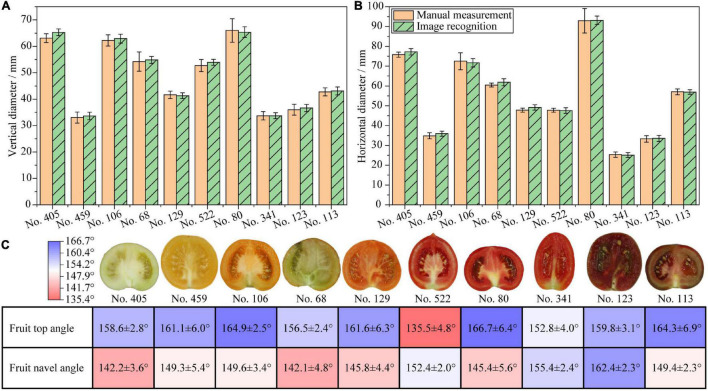
Verification of phenotyping indicators from fruit vertical cut images. **(A)** Vertical diameters of tomato fruits; **(B)** Horizontal diameters of tomato fruits; **(C)** Fruit top and navel angles, fruit images are not proportional.

The measurements of the tomato fruit vertical diameter and horizontal diameter were verified. The results are shown in Figures 3A,B. The results show that the measurements based on image recognition accord with the manual measurements. No. 80 has the largest horizontal and vertical diameter. No. 341 and No. 459 have the shortest horizontal and vertical diameter, respectively. The vertical diameter of No. 341 and No. 459 are quite close, which accord with the proportional images in Figure 1A. Significance tests suggest that there are no significant differences between the results of image recognition and manual measurements in the same sample. The RMSE values of the vertical and horizontal diameter measurements are 0.016 and 0.017, respectively, which are less than 0.100, an empirical threshold of RMSE determining the accuracy of measurement results. In addition, the Pearson’s correlation *R*^2^ between the image recognition and the manual measurements is greater than 0.99. These indicate that the accuracy of the image recognition measurement is equivalent to that of manual measurement. The unequal variance test indicates homogeneous variances between the results of the manual measurement and the image recognition (*p* = 0.265), indicating that the variances between image recognition and manual measurements are equal. This implies that the fruit vertical and horizontal diameter measurements by image recognition are as precise as manual measurements.

The results of fruit top and navel angles are shown in Figure 3C. Large fruit top angles indicate flat fruit top shape, whereas small fruit top angles indicate sharp protruding fruit top shape. Large fruit navel angles indicate flat fruit navel shape, whereas small fruit navel angles indicate deep indented or concave fruit navel shape. Among all samples, No. 522 has the smallest fruit top angle, which is due to the sharp protrusion on the fruit topside. No. 80 has the largest fruit top angle in accordance with the smooth and flat shape on the fruit topside. Fruit top angles of all samples except No. 522 are larger than 150°, which accord with the sensory judgment. No. 405 has the smallest fruit navel angle among all samples as the indentation on the fruit navel side is quite deep. No. 123 has the largest fruit navel angle as the indentation on the fruit navel side is quite shallow. For most samples, the fruit navel angles are smaller than the fruit top angles as expected, except that No. 522, 341, and 123 show the opposite pattern. Although the fruit top and navel angles are difficult to measure manually, the results accord quite close with the sensory judgment. Therefore, the fruit top and navel angles by image recognition are feasible to reflect the tomato fruit top and navel phenotypes quantitatively. Detailed fruit lengths and angles data are listed in Supplementary Table 1.

### Performance of Phenotyping From Horizontal Cut Images

The number of iterations has an impact on the training results of deep learning models. The training and validation datasets had closely identical variation trends, indicating that the model was not overfitted with the parameters chosen during the validation procedure. The loss value reduces as the number of iterations increases, but it remains rather consistent as the number of iterations hits 4,000, and it progressively approaches the minimum value of 0.1505. The results show that the Mask R-CNN used in this model can learn the features efficiently and converge rapidly, indicating that it has the potential to accomplish the required objectives. The detailed loss curves of the training and validation sets for 5,000 iterations are shown in Supplementary Figure 1A.

When evaluating the performance of a model, both recall and precision are critical. As recall increases, the precision decreases, but the precision of an outperforming model keeps a high level as recall increases, implying the model will segment a large majority of TP before detecting FP. Thus, the *F*1*-score*, the harmonic average of precision and recall, is used to evaluate the model performance. Values between 0 and 1 are set as various thresholds. Segmentations having a prediction probability larger than the threshold are considered positive. The *F*1*-score* reaches the maximum of 0.8620 when the threshold is 0.6, implying that 0.6 is a trade-off that balances the precision and recall and maximizes the performance of the model. The AP is 0.8753, implying that the segmented locule areas match the true locule areas more than 87.53% of the circumstances. When the threshold is 0.9, the AP is 0.8107 (greater than 0.8). This indicates that the model based on Mask R-CNN has high accuracy. The trajectories of the *F*1*-score* at various thresholds are shown in Supplementary Figure 1B.

**FIGURE 4:**
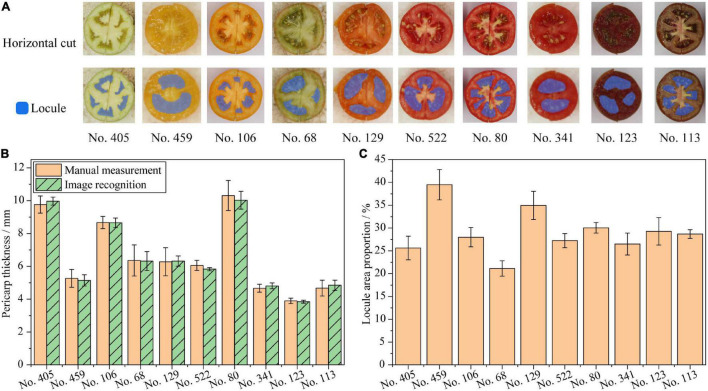
Verification of phenotyping indicators from fruit horizontal cut images. **(A)** Fruit locule segmentation, fruit images are not proportional; **(B)** Pericarp thickness of tomato fruits; **(C)** Locule area proportions of tomato fruits.

The measurements of tomato fruit locule number and pericarp thickness were verified and presented in Figure 4. Blue regions represent the locule segmentation by the deep learning segmentation model. The results suggest that the model can accurately segment locule areas from the fruit horizontal cut images after training. Color, fruit size, and seeds have barely influenced the model segmentation performance. No. 106 and No. 80 in Figure 4A indicate that small locules which are not fully formed in the fruit can also be segmented by the model. The locule counting results in Table 2 show that locule counted by the deep learning segmentation model is promising, suggesting that the model can automatically count the fruit locule number with perfect accuracy. The only wrongly counted cultivar among all samples is No. 459, which is also difficult for manual counting due to its small fruit size and hazy septum.

**TABLE 2:** Tomato samples for image acquisition and phenotyping verification.

Cultivar no.	Average locule number per fruit^a^	
	Manual count	Deep learning segmentation
No. 405	4.25	4.25
No. 459	1.50	1.25
No. 106	3.50	3.50
No. 68	2.50	2.50
No. 129	3.00	3.00
No. 522	2.50	2.50
No. 80	4.50	4.50
No. 341	2.00	2.00
No. 123	2.50	2.50
No. 113	4.50	4.50

*^a^The averages are based on all tomato fruits in one horizontal cut image.*

The results in Figure 4B show that the pericarp thickness measurements based on image recognition accord with the manual measurements. No. 80 and No. 123 have the thickest and thinnest fruit pericarp, respectively. The thickness of No. 68 and No. 129 are quite close in accordance with the sensory judgment. Significance tests suggest that there are no significant differences between the pericarp thickness by image recognition and manual measurements in the same sample. The RMSE of the pericarp thickness measurement is 0.024, which is less than 0.100, indicating high accuracy of image recognition measurement. In addition, the Pearson’s correlation *R*^2^ between the image recognition and the manual measurements is greater than 0.99. This indicates that the accuracy of the pericarp thickness measurement by image recognition is equivalent to that of manual measurement. The unequal variance test indicates homogeneous variances between the results of the manual measurement and the image recognition (*p* = 0.303), indicating that the variances between image recognition and manual measurements are equal. This implies that the pericarp thickness measurements by image recognition are as precise as manual measurements.

The results of fruit locule area proportion are shown in Figure 4C. A larger locule area proportion indicates a larger locule volume proportion in the fruit. Among all samples, No. 459 and No. 68 have the largest and smallest locule area proportion, respectively. No. 129 has a larger locule area proportion than No. 106. The locule area proportions of No. 522 and No. 341 are quite close. These all accord with the sensory judgment from the fruit images. Although the fruit locule area proportions are difficult to measure manually, the results accord quite close with the sensory judgment. Therefore, the fruit locule area proportions measured by the deep learning segmentation model can quantitatively represent tomato fruit locule phenotypes. Detailed fruit locule number, locule area proportion, and pericarp thickness data are listed in Supplementary Table 1.

## Discussion

This study proposed a combination of image recognition and deep learning segmentation methods to extract several important tomato fruit phenotypes quantitatively. The manual measuring of these phenotypes is essential for tomato breeding but is time-consuming. The proposed combination of methods can reduce the operating steps of tomato fruit phenotyping and increase the measurement accuracy, which greatly reduces the phenotyping time consumption. The HSV filtering and binarization process is applicable to isolate most pure color and black tomato fruits from the white background with a respective combination of S and V thresholds, which meets the requirement in most cases. Mottled tomato fruits use a different combination of S and V thresholds, but such cultivars are uncommon. Considering black tomato fruits do not fit the black background, blue might be a more general background color for tomato fruit phenotyping because blue or similar colored fruits are not found among all cultivars in this study or other studies (Merk et al., 2012). In addition, the images of intact tomato fruits are taken from the navel side instead of from the top side. This is because the shape, size, and other aspects of the sepals are also important phenotypes that require further analysis. The navel side images of intact tomato fruits include both extractable fruit and sepal phenotypes, which could investigate more phenotypes without increasing the number of images per tomato cultivar.

**FIGURE 5:**
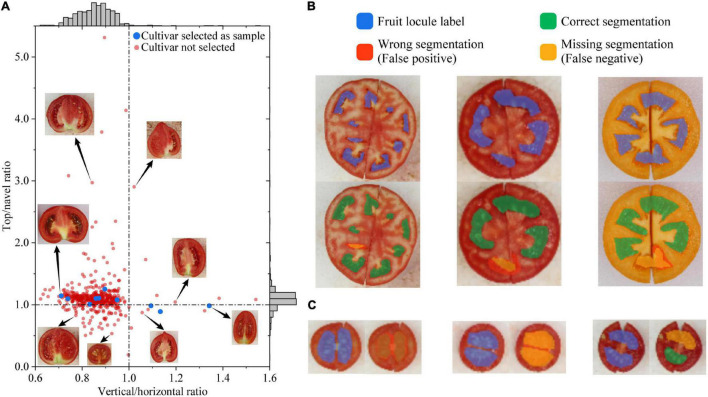
Evaluation of tomato fruit phenotyping indicators and fruit locule segmentation. **(A)** Distribution of top/navel ratio against horizontal/vertical ratio among cultivars; the dashed line refers to the corresponding value equals one; **(B)** Incorrect segmentation cases that do the correct locule counting; **(C)** Incorrect segmentation cases caused by hazy septum. Fruit images are not proportional.

Based on the horizontal and vertical diameter and the navel and top angle, their corresponding ratios are also important fruit phenotypes, i.e., vertical/horizontal ratio and top/navel ratio. All cultivars are plotted with the top/navel ratio at the vertical axis and the vertical/horizontal ratio at the horizontal axis in Figure 5A. Most cultivars have a top/navel ratio between 0.9 and 1.3 and also a vertical/horizontal ratio between 0.7 and 1.0, indicating most tomato fruits have a longer horizontal diameter and a larger top angle. Although all samples have a linear trend in the plot (*R*^2^ = 0.6995), all cultivars do not fit a specific trend, indicating that the top/navel ratio does not correlate with the vertical/horizontal ratio. Several cultivars are shown in Figure 5A to represent the sensory differences.

To increase locule segmentation performance, the deep learning model applied the feature mapping strategy (Gupta, 2014) to merge color and texture features in the FPN structure when training the Mask R-CNN. In this study, tomato fruits with different colors, sizes, and locule shapes were tested to evaluate the locule segmentation performance of the deep learning model. The tomato fruit locules are separately located on one surface in the images, thus overlapping and occluding do not occur in these scenarios. However, the deep learning model recognizes mainly two types of incorrect segmentations: ones that do the correct locule counting and ones that are caused by a hazy septum (Figures 5B,C). In the first type, one obstacle to locule segmentation is the slit between two joint parts of fruit cut. As shown in Figure 4A, although the fruit parts are joined together before taking the horizontal cut images, the slit between two joint parts does not influence the segmentation performance. Still, the only incorrect segmentation caused by the slit is shown on the right in Figure 5B, where the slit has passed through the locule. This implies that the fruit parts should be joined together closely or cut another fruit before taking the horizontal cut images. Figure 5B left and middle also shows representative incorrect segmentations that are partly correct and do the correct counting. Such cases do not miss any locules but still be recognized as missing segmentation due to the low IoU, yet they should be considered as correct in practice (Tripodi et al., 2018). In the second type, the most common incorrect segmentation cases are shown in Figure 5C. The tomato fruits in these cases all have small horizontal diameters and hazy septum. Manual labeling is difficult and unreliable for these cases, which could be the reason for wrong and missing segmentations. In practice, the locule number of such fruit is not essential and could be considered as one, whereas the recognition results of locule area proportion and pericarp thickness could be considered correct. With the abovementioned correct counting cases (except Figure 5B right) and hazy septum cases modified as correct, the AP is 0.9564 when the threshold is 0.6, indicating that the model is quite promising for tomato fruit locule segmentation and outperforms other image recognition methods. The results also indicate that the model is robust to segment fruit locules with different colors, sizes, and locule shapes. Compared with *Tomato Analyzer* (Gonzalo et al., 2009) and *LocAnalyzer* (Spetale et al., 2020), the advantage of the proposed methods is the ability to measure pericarp thickness, locule number, and area automatically. Moreover, the locule segmentation model increases the phenotyping efficiency by processing multi-fruit images instead of scanning one fruit each time, while still having a similar or even higher AP than *LocAnalyzer*.

The collection and analysis of phenotypes in a consistent manner is requisite for plant phenomics research (Song et al., 2021). Current crop phenotyping systems apply high-throughput techniques to capture numerous phenotypes automatically, and the accuracy of these procedures is steadily increasing (Zhang et al., 2021). The proposed combination of methods can automatically collect a group of tomato fruit phenotypes from a set of tomato fruit images. The results of the verification indicate that the locule number is reliably counted, and the locule area proportion is quantitatively defined. The fruit horizontal and vertical diameter, top and navel angle, and pericarp thickness are also accurately recognized. Furthermore, the proposed combination of methods may considerably minimize manual observation workloads and make cultivar investigation during tomato breeding more time-efficient. Operation mistakes during manual measurement can also be prevented. Besides, the efficiency and accuracy during the investigation of tomato fruit phenotypes are greatly improved, particularly when assessing fruit locule number and area proportion.

In terms of the image acquisition method, all the fruit images are taken with a fixed focal length in a constant illumination condition, and the color space values are utilized to describe the fruit color, allowing the tomato fruit color to be shown quantitatively under the same standard. Nevertheless, the juice of the tomato fruit cut parts is likely to contaminate the background in practice, which requires cleaning up before taking each image. Based on the phenotyping demand and barriers in tomato breeding, this study proposed a quantitative and high-throughput phenotyping tool that integrates fruit processing, image acquisition, and phenotypes extraction. The tool is proved feasible and promising and has significant implications for the phenotypes evaluation of tomato fruit (Kim et al., 2021). Since tomato is the model species of fruit development studies, the methodology could also be extended to other fruit crops.

It is important for omics research to assess the tomato fruit phenotypes with a consistent and quantitative phenotyping tool based on image recognition and deep learning segmentation. Such consistent and quantitative phenotype data could also aid tomato genomic, metabolomic, and transcriptomic investigations. For example, studies have found several QTLs that control tomato fruit morphological shape and locule number (Illa-Berenguer et al., 2015; Barraj et al., 2021). Quantitative phenotyping tools also allow researchers to delve deeper into the mechanisms behind the formation of certain traits in tomato fruit at the genetic level (Marefatzadeh-Khameneh et al., 2021). Research on the association network analysis between tomato fruit color and metabolic pathways would possibly be accelerated further as well (Hu et al., 2020; Yuan et al., 2021).

## Conclusion

In this study, a combination of image recognition and deep learning model is proposed to extract tomato fruit phenotypes quantitatively and automatically. First, images of intact tomatoes, vertical cut fruits, and horizontal cut fruits were acquired under a constant illumination condition. Second, the method based on image recognition isolated fruits from the background and extract fruit color, vertical/horizontal diameters, and top/navel angles from the images of intact tomatoes and vertical cut. Finally, the deep learning model based on Mask R-CNN was trained and tested to segment locules from the images of horizontal cut. The locule number, locule area proportion, and pericarp thickness were thus extracted automatically. The proposed combination of methods improves the efficiency and accuracy of tomato fruit phenotyping. The proposed deep learning model segments tomato fruit locules with high average precision, implying that the whole combination of methods is a promising tool to evaluate tomato fruit phenotypes thoroughly. In conclusion, the results of this study provide technical support for the quantitative analysis and evaluation of tomato fruit phenotyping, which is important for tomato breeding. Furthermore, the methodology could be extended to the phenotyping of other fruit crops.

## Data Availability Statement

The original contributions presented in the study are included in the article/Supplementary Material, further inquiries can be directed to the corresponding authors.

## Author Contributions

YZu and YC conceived and designed the concept and methodology. HW, RW, and YC prepared the tomato fruits and other materials. QG, YZa, and YC conducted the experiments. YZu and XZ realized the algorithms and methods, analyzed the data, and prepared the figures and tables. YZu wrote the manuscript. YC and XZ supervised the manuscript and made valuable inputs. All authors read and approved the submission of the manuscript.

## Conflict of Interest

The authors declare that the research was conducted in the absence of any commercial or financial relationships that could be construed as a potential conflict of interest.

## Publisher’s Note

All claims expressed in this article are solely those of the authors and do not necessarily represent those of their affiliated organizations, or those of the publisher, the editors and the reviewers. Any product that may be evaluated in this article, or claim that may be made by its manufacturer, is not guaranteed or endorsed by the publisher.
