# Supplementary material for: Quantitative Extraction and Evaluation of Tomato Fruit Phenotypes Based on Image Recognition
Source: Front Plant Sci. 2022 Apr 13;13:859290. doi: 10.3389/fpls.2022.859290 (PMC9044966; doi:10.3389/fpls.2022.859290)
Supplement: Supplementary file 1 [file Data_Sheet_1.DOCX]

Code

**used_codes.cs |** C# code for the methods.

**using System;**

**using System.Collections.Generic;**

**using System.ComponentModel;**

**using System.Data;**

**using System.Drawing;**

**using System.Linq;**

**using System.Text;**

**using System.Windows.Forms;**

**using Emgu.CV;**

**using Emgu.CV.Structure;**

**using Emgu.CV.CvEnum;**

**using ImageWork;**

**using System.Drawing.Imaging;**

**using System.Collections;**

**using System.Data.OleDb;**

**using System.IO;**

**using pzcom;**

**using Microsoft.Win32;**

**using DirectShowLib;**

**using EDSDKLib;**

**using System.Runtime.InteropServices;**

**using ZXing;**

**using System.Text.RegularExpressions;**

**using System.Net;**

**using Newtonsoft.Json;**

**namespace LeafWork**

**{**

**public partial class Form1 : Form**

**{**

**public Form1()**

**{**

**InitializeComponent();**

**CameraHandler = new SDKHandler();**

**CameraHandler.CameraAdded += new SDKHandler.CameraAddedHandler(SDK_CameraAdded);**

**CameraHandler.SDKObjectEvent += new EDSDK.EdsObjectEventHandler(Camera_SDKObjectEvent);**

**CameraHandler.LiveViewUpdated += new SDKHandler.ImageUpdate(SDK_LiveViewUpdated);**

**}**

**DsDevice[] _SystemCamereas;**

**SDKHandler CameraHandler;**

**List<int> AvList;**

**List<int> TvList;**

**List<int> ISOList;**

**List<Camera> CamList;**

**private double ratio = 1;**

**private bool landscape = true,inline=false;**

**private int rectl, rectt, rectw, recth;**

**string AppPath="",workdir = "";**

**BarcodeReader reader = new BarcodeReader();**

**string[] rgb = new string[147];**

**static string Read1(string filename)**

**{**

**BarcodeReader reader = new BarcodeReader();**

**reader.Options.CharacterSet = "UTF-8";**

**Bitmap map = new Bitmap(filename);**

**Result result = reader.Decode(map);**

**return result == null ? "" : result.Text;**

**}**

**private void SDK_CameraAdded()**

**{**

**RefreshCamera();**

**}**

**private void CloseSession()**

**{**

**CameraHandler.CloseSession();**

**camAV.Items.Clear();**

**camTV.Items.Clear();**

**camISO.Items.Clear();**

**camcolort.Enabled = true;**

**camWB.Enabled = true;**

**camsetting.Enabled = false;**

**}**

**private void RefreshCamera()**

**{**

**CloseSession();**

**camlist.Items.Clear();**

**CamList = CameraHandler.GetCameraList();**

**foreach (Camera cam in CamList) camlist.Items.Add(cam.Info.szDeviceDescription);**

**if (CamList.Count > 0) camlist.SelectedIndex = 0;**

**}**

**private void SDK_LiveViewUpdated(System.Drawing.Image img)**

**{**

**try**

**{**

**if (CameraHandler.IsLiveViewOn)**

**{**

**if (cc.checkReg())**

**{**

**Graphics g = Graphics.FromImage(img);**

**int hintw = 150;**

**g.DrawString(this.Text, new Font("Arial", 12f, FontStyle.Bold), new SolidBrush(Color.Gray), img.Width / 2 - hintw, img.Height / 2);**

**g.DrawString(this.Text, new Font("Arial", 12f, FontStyle.Bold), new SolidBrush(Color.Gray), img.Width / 4 - hintw, img.Height / 4);**

**g.DrawString(this.Text, new Font("Arial", 12f, FontStyle.Bold), new SolidBrush(Color.Gray), img.Width * 3 / 4 - hintw, img.Height / 4);**

**g.DrawString(this.Text, new Font("Arial", 12f, FontStyle.Bold), new SolidBrush(Color.Gray), img.Width / 4 - hintw, img.Height * 3 / 4);**

**g.DrawString(this.Text, new Font("Arial", 12f, FontStyle.Bold), new SolidBrush(Color.Gray), img.Width * 3 / 4 - hintw, img.Height * 3 / 4);**

**g.Dispose();**

**}**

**imgpre.Image = img;**

**Result res = reader.Decode(new Bitmap(img));**

**if (res.Text.Split('-').Length >= 2)**

**{**

**if (imgid.Text != res.Text)**

**{**

**imgid.Text = res.Text;**

**if (istakephoto.Checked)**

**{**

**inline = true;**

**CameraHandler.TakePhoto();**

**}**

**else**

**{**

**new Bitmap(img).Save(workdir + imgid.Text + "_" + DateTime.Now.ToString("yyyyMMddhhmmss") + ".jpg", ImageFormat.Jpeg);**

**}**

**}**

**}**

**}**

**}**

**catch { }**

**}**

**private void calRatio(System.Drawing.Image actual)**

**{**

**landscape = (Convert.ToDouble(imgpre.Width) / imgpre.Height <= Convert.ToDouble(actual.Width) / actual.Height);**

**if (landscape)**

**ratio = Convert.ToDouble(actual.Width) / imgpre.Width;**

**else**

**ratio = Convert.ToDouble(actual.Height) / imgpre.Height;**

**if (landscape)**

**{**

**rectl = Convert.ToInt16(m_ptStart.X * ratio);**

**rectt = Convert.ToInt16((m_ptStart.Y - (imgpre.Height - actual.Height / ratio) / 2) * ratio);**

**}**

**else**

**{**

**rectl = Convert.ToInt16((m_ptStart.X - (imgpre.Width - actual.Width / ratio) / 2) * ratio);**

**rectt = Convert.ToInt16(m_ptStart.Y * ratio);**

**}**

**}**

**private uint Camera_SDKObjectEvent(uint inEvent, IntPtr inRef, IntPtr inContext)**

**{**

**switch (inEvent)**

**{**

**case EDSDK.ObjectEvent_All:**

**break;**

**case EDSDK.ObjectEvent_DirItemRequestTransfer: if (CameraHandler.CameraPIC != null)**

**{**

**try**

**{**

**calRatio(CameraHandler.CameraPIC);**

**rectw = Convert.ToInt16((m_ptEnd.X - m_ptStart.X) * ratio);**

**recth = Convert.ToInt16((m_ptEnd.Y - m_ptStart.Y) * ratio);**

**if (rectw > 0 && recth > 0)**

**{**

**Rectangle rect = new Rectangle(rectl, rectt, rectw, recth);**

**imageSource = new Image<Bgr, byte>(new Bitmap(CameraHandler.CameraPIC));**

**imageSource = imageSource.GetSubRect(rect);**

**CameraHandler.CameraPIC = imageSource.ToBitmap();**

**}**

**if (cc.checkReg())**

**{**

**Graphics g = Graphics.FromImage(CameraHandler.CameraPIC);**

**int x = 5, y = 10;**

**for (int j = 0; j < y; j++)**

**for (int i = 0; i < x; i++)**

**{**

**g.DrawString(this.Text, new Font("Arial", 15f, FontStyle.Bold), new SolidBrush(Color.Gray), CameraHandler.CameraPIC.Width * i / x, CameraHandler.CameraPIC.Height * j / y);**

**imgpre.Image = CameraHandler.CameraPIC;**

**origin = CameraHandler.CameraPIC;**

**if (inline)**

**{**

**inline = false;**

**imgpre.Image.Save(workdir + imgid.Text + "_" + DateTime.Now.ToString("yyyyMMddhhmmss") + ".jpg", ImageFormat.Jpeg);**

**}**

**}**

**catch (Exception err) { MessageBox.Show("发现错误:"+err.Message); }**

**}**

**break;**

**}**

**return EDSDK.EDS_ERR_OK;**

**}**

**private void camAV_SelectedIndexChanged(object sender, EventArgs e)**

**{**

**CameraHandler.SetSetting(EDSDK.PropID_Av, CameraValues.AV((string)camAV.SelectedItem));**

**}**

**private void camTV_SelectedIndexChanged(object sender, EventArgs e)**

**{**

**CameraHandler.SetSetting(EDSDK.PropID_Tv, CameraValues.TV((string)camTV.SelectedItem));**

**}**

**private void camISO_SelectedIndexChanged(object sender, EventArgs e)**

**{**

**CameraHandler.SetSetting(EDSDK.PropID_ISOSpeed, CameraValues.ISO((string)camISO.SelectedItem));**

**}**

**private void camOpen_Click(object sender, EventArgs e)**

**{**

**if (CameraHandler.CameraSessionOpen)**

**{**

**RefreshCamera();**

**camOpen.Text = "连接";**

**liveview.Enabled = false;**

**camSnap.Enabled = false;**

**}**

**else OpenSession();**

**}**

**private void OpenSession()**

**{**

**if (camlist.SelectedIndex >= 0)**

**{**

**try**

**{**

**CameraHandler.ImageSaveDirectory = workdir;**

**CameraHandler.OpenSession(CamList[camlist.SelectedIndex]);**

**string cameraname = CameraHandler.MainCamera.Info.szDeviceDescription;**

**saveraw.Checked = false;**

**saveraw.Enabled = true;**

**if (CameraHandler.GetSetting(EDSDK.PropID_AEMode) != EDSDK.AEMode_Mamual)**

**{**

**saveraw.Enabled = false;**

**MessageBox.Show("相机处于非手动模式,拍摄参数设置无法使用!", "提示");**

**}**

**AvList = CameraHandler.GetSettingsList((uint)EDSDK.PropID_Av);**

**TvList = CameraHandler.GetSettingsList((uint)EDSDK.PropID_Tv);**

**ISOList = CameraHandler.GetSettingsList((uint)EDSDK.PropID_ISOSpeed);**

**foreach (int Av in AvList) camAV.Items.Add(CameraValues.AV((uint)Av));**

**foreach (int Tv in TvList) camTV.Items.Add(CameraValues.TV((uint)Tv));**

**foreach (int ISO in ISOList) camISO.Items.Add(CameraValues.ISO((uint)ISO));**

**camAV.SelectedIndex = camAV.Items.IndexOf(CameraValues.AV((uint)CameraHandler.GetSetting((uint)EDSDK.PropID_Av)));**

**camTV.SelectedIndex = camTV.Items.IndexOf(CameraValues.TV((uint)CameraHandler.GetSetting((uint)EDSDK.PropID_Tv)));**

**camISO.SelectedIndex = camISO.Items.IndexOf(CameraValues.ISO((uint)CameraHandler.GetSetting((uint)EDSDK.PropID_ISOSpeed)));**

**if (cameraname != "Canon EOS 1100D" && cameraname != "Canon EOS Rebel T3" && cameraname != "Canon EOS Kiss X50")**

**{**

**int wbidx = (int)CameraHandler.GetSetting((uint)EDSDK.PropID_WhiteBalance);**

**camWB.SelectedIndex = (wbidx > 8) ? wbidx - 1 : wbidx;**

**}**

**else**

**{**

**camcolort.Enabled = false;**

**camWB.Enabled = false;**

**}**

**camsetting.Enabled = true;**

**CameraHandler.SetSetting(EDSDK.PropID_SaveTo, (uint)EDSDK.EdsSaveTo.Host);**

**CameraHandler.SetCapacity();**

**camImage.SelectedIndex = 1;**

**CameraHandler.SetSetting(EDSDK.PropID_ImageQuality, (uint)EDSDKLib.EDSDK.ImageQuality.EdsImageQuality_MJF);**

**afmode.SelectedIndex = 0;**

**CameraHandler.SetSetting(EDSDK.PropID_AFMode, (uint)afmode.SelectedIndex);**

**camOpen.Text = "断开";**

**liveview.Enabled = true;**

**camSnap.Enabled = true;**

**}**

**catch (Exception err)**

**{**

**MessageBox.Show(err.Message);**

**}**

**}**

**}**

**private void liveview_Click(object sender, EventArgs e)**

**{**

**try**

**{**

**if (!CameraHandler.IsEvfFilming)**

**{**

**if (!CameraHandler.IsLiveViewOn)**

**{**

**imgpre.Dock = DockStyle.Fill;**

**imgpre.SizeMode = PictureBoxSizeMode.Zoom;**

**inthumb.Checked = true;**

**CameraHandler.StartLiveView();**

**canonpanel.Height = 50;**

**liveview.Text = "停止";**

**}**

**else**

**{**

**CameraHandler.StopLiveView();**

**canonpanel.Height = 520;**

**liveview.Text = "预览";**

**}**

**canonpanel.Tag = canonpanel.Width + ":" + canonpanel.Height + ":" + canonpanel.Left + ":" + canonpanel.Top + ":" + canonpanel.Font.Size;**

**FocusFar2Button.Enabled = (liveview.Text != "预览");**

**FocusNear2Button.Enabled = (liveview.Text != "预览");**

**}**

**}**

**catch (Exception err)**

**{**

**MessageBox.Show(err.Message, "错误");**

**}**

**}**

**private void camSnap_Click(object sender, EventArgs e)**

**{**

**try**

**{**

**if (CameraHandler.IsLiveViewOn)**

**{**

**CameraHandler.StopLiveView();**

**liveview.Text = "预览";**

**Application.DoEvents();**

**}**

**CameraHandler.TakePhoto();**

**}**

**catch (Exception err)**

**{**

**MessageBox.Show(err.Message, "错误");**

**}**

**}**

**private void save2cam_CheckedChanged(object sender, EventArgs e)**

**{**

**if (save2cam.Checked)**

**CameraHandler.SetSetting(EDSDK.PropID_SaveTo, (uint)EDSDK.EdsSaveTo.Both);**

**else**

**CameraHandler.SetSetting(EDSDK.PropID_SaveTo, (uint)EDSDK.EdsSaveTo.Host);**

**CameraHandler.SetCapacity();**

**}**

**private void camImage_SelectedIndexChanged(object sender, EventArgs e)**

**{**

**if (saveraw.Checked)**

**{**

**if (camImage.Text == "高")**

**CameraHandler.SetSetting(EDSDK.PropID_ImageQuality, (uint)EDSDKLib.EDSDK.ImageQuality.EdsImageQuality_LRLJF);**

**else if (camImage.Text == "中")**

**CameraHandler.SetSetting(EDSDK.PropID_ImageQuality, (uint)EDSDKLib.EDSDK.ImageQuality.EdsImageQuality_LRMJF);**

**else**

**CameraHandler.SetSetting(EDSDK.PropID_ImageQuality, (uint)EDSDKLib.EDSDK.ImageQuality.EdsImageQuality_LRSJF);**

**}**

**else**

**{**

**if (camImage.Text == "高")**

**CameraHandler.SetSetting(EDSDK.PropID_ImageQuality, (uint)EDSDKLib.EDSDK.ImageQuality.EdsImageQuality_LJF);**

**else if (camImage.Text == "中")**

**CameraHandler.SetSetting(EDSDK.PropID_ImageQuality, (uint)EDSDKLib.EDSDK.ImageQuality.EdsImageQuality_MJF);**

**else**

**CameraHandler.SetSetting(EDSDK.PropID_ImageQuality, (uint)EDSDKLib.EDSDK.ImageQuality.EdsImageQuality_SJF);**

**}**

**}**

**private void camcolort_ValueChanged(object sender, EventArgs e)**

**{**

**CameraHandler.SetSetting(EDSDK.PropID_ColorTemperature, (uint)camcolort.Value);**

**}**

**private void camWB_SelectedIndexChanged(object sender, EventArgs e)**

**{**

**switch (camWB.SelectedIndex)**

**{**

**case 0: CameraHandler.SetSetting(EDSDK.PropID_WhiteBalance, EDSDK.WhiteBalance_Auto); break;**

**case 1: CameraHandler.SetSetting(EDSDK.PropID_WhiteBalance, EDSDK.WhiteBalance_Daylight); break;**

**case 2: CameraHandler.SetSetting(EDSDK.PropID_WhiteBalance, EDSDK.WhiteBalance_Cloudy); break;**

**case 3: CameraHandler.SetSetting(EDSDK.PropID_WhiteBalance, EDSDK.WhiteBalance_Tangsten); break;**

**case 4: CameraHandler.SetSetting(EDSDK.PropID_WhiteBalance, EDSDK.WhiteBalance_Fluorescent); break;**

**case 5: CameraHandler.SetSetting(EDSDK.PropID_WhiteBalance, EDSDK.WhiteBalance_Strobe); break;**

**case 6: CameraHandler.SetSetting(EDSDK.PropID_WhiteBalance, EDSDK.WhiteBalance_WhitePaper); break;**

**case 7: CameraHandler.SetSetting(EDSDK.PropID_WhiteBalance, EDSDK.WhiteBalance_Shade); break;**

**case 8: CameraHandler.SetSetting(EDSDK.PropID_WhiteBalance, EDSDK.WhiteBalance_ColorTemp); break;**

**case 9: CameraHandler.SetSetting(EDSDK.PropID_WhiteBalance, EDSDK.WhiteBalance_PCSet1); break;**

**case 10: CameraHandler.SetSetting(EDSDK.PropID_WhiteBalance, EDSDK.WhiteBalance_PCSet2); break;**

**case 11: CameraHandler.SetSetting(EDSDK.PropID_WhiteBalance, EDSDK.WhiteBalance_PCSet3); break;**

**}**

**if (camWB.SelectedIndex == 8) camcolort.Enabled = true;**

**else camcolort.Enabled = false;**

**}**

**private void afmode_SelectedIndexChanged(object sender, EventArgs e)**

**{**

**CameraHandler.SetSetting(EDSDK.PropID_AFMode, (uint)afmode.SelectedIndex);**

**}**

**private void tabPage1_DragEnter(object sender, DragEventArgs e)**

**private void inthumb_Click(object sender, EventArgs e)**

**{**

**if (inthumb.Checked)**

**{**

**imgpre.Dock = DockStyle.Fill;**

**imgpre.SizeMode = PictureBoxSizeMode.Zoom;**

**}**

**else**

**{**

**imgpre.Dock = DockStyle.None;**

**imgpre.SizeMode = PictureBoxSizeMode.AutoSize;**

**}**

**}**

**private System.Drawing.Point m_ptStart = new System.Drawing.Point(0, 0);**

**private System.Drawing.Point m_ptEnd = new System.Drawing.Point(0, 0);**

**private System.Drawing.Point mm = new System.Drawing.Point(0, 0);**

**private bool m_bMouseDown = false;**

**private int imgW = 0, imgH = 0, imgtop = 0, imgleft = 0;**

**private Emgu.CV.Capture _capture = null;**

**private delegate void DisplayImageDelegate(Bitmap Image);**

**private void DisplayImage(Bitmap Image)**

**{**

**if (imgpre.InvokeRequired)**

**{**

**try**

**{**

**DisplayImageDelegate DI = new DisplayImageDelegate(DisplayImage);**

**this.BeginInvoke(DI, new object[] { Image });**

**}**

**catch (Exception ex)**

**{**

**}**

**}**

**else**

**{**

**imgpre.Image = Image;**

**imgpre.Refresh();**

**}**

**}**

**private void imgpre_Paint(object sender, PaintEventArgs e)**

**{**

**if (getebv.Checked || getbkv.Checked || getresolution.Checked)**

**{**

**e.Graphics.DrawRectangle(System.Drawing.Pens.Green, new Rectangle(mm.X, mm.Y, cr, cr));**

**}**

**else**

**{**

**if (autolength.Checked||drawwhiteline.Checked)**

**{**

**Pen pn = new Pen(Color.White, 2.0f);**

**e.Graphics.DrawLine(pn, m_ptStart, m_ptEnd);**

**if (lp.Count > 1)**

**e.Graphics.DrawLines(pn, lp.ToArray());**

**}**

**else**

**{**

**e.Graphics.DrawRectangle(System.Drawing.Pens.Red,**

**new Rectangle(m_ptStart.X, m_ptStart.Y, m_ptEnd.X - m_ptStart.X, m_ptEnd.Y - m_ptStart.Y));**

**imgW = Convert.ToInt16((m_ptEnd.X - m_ptStart.X) * ratio);**

**imgH = Convert.ToInt16((m_ptEnd.Y - m_ptStart.Y) * ratio);**

**}**

**}**

**if (liveview.Text == "停止" && camOpen.Text == "断开")**

**if (cankwx.Checked)**

**{**

**for (int i = 1; i < lineh.Value + 1; i++)**

**{**

**int x = Convert.ToInt16(imgpre.Height * i / (lineh.Value + 1));**

**e.Graphics.DrawLine(System.Drawing.Pens.Gray, 0, x, imgpre.Width, x);**

**}**

**for (int i = 1; i < lines.Value + 1; i++)**

**{**

**int x = Convert.ToInt16(imgpre.Width * i / (lines.Value + 1));**

**e.Graphics.DrawLine(System.Drawing.Pens.Gray, x, 0, x, imgpre.Height);**

**}**

**int dx = imgpre.Width / 2;**

**int dy = imgpre.Height / 2;**

**e.Graphics.DrawLine(System.Drawing.Pens.Yellow, dx, dy - 8, dx, dy + 8);**

**e.Graphics.DrawLine(System.Drawing.Pens.Yellow, dx - 8, dy, dx + 8, dy);**

**}**

**}**

**}**

**private void imgpre_MouseMove(object sender, MouseEventArgs e)**

**{**

**if (autolength.Checked || drawwhiteline.Checked) this.Cursor = Cursors.Cross;**

**else this.Cursor = Cursors.Default;**

**mm = new System.Drawing.Point(e.X - cr / 2, e.Y - cr / 2);**

**imgpre.Refresh();**

**if (!m_bMouseDown) return;**

**m_ptEnd = new System.Drawing.Point(e.X, e.Y);**

**}**

**private void imgpre_MouseUp(object sender, MouseEventArgs e)**

**{**

**if (!autolength.Checked && !drawwhiteline.Checked)**

**{**

**m_bMouseDown = !m_bMouseDown;**

**imgpre.Refresh();**

**imgpre.Focus();**

**}**

**}**

**private void imgpre_MouseDown(object sender, MouseEventArgs e)**

**{**

**if (e.Button == MouseButtons.Left)**

**{**

**if (autolength.Checked || drawwhiteline.Checked)**

**{**

**if (e.Button == MouseButtons.Right)**

**{**

**lp.Clear();**

**m_bMouseDown = false;**

**}**

**else**

**{**

**if (!m_bMouseDown)**

**{**

**changd = 0;**

**lp.Clear();**

**m_ptStart = new System.Drawing.Point(e.X, e.Y);**

**m_ptEnd = new System.Drawing.Point(e.X, e.Y);**

**lp.Add(m_ptStart);**

**m_bMouseDown = !m_bMouseDown;**

**}**

**else**

**{**

**double mmperdot = 25.4 / Convert.ToDouble(imgdpi.Value);**

**changd = changd + (Math.Sqrt((Math.Pow(m_ptStart.Y - m_ptEnd.Y, 2) + Math.Pow(m_ptStart.X - m_ptEnd.X, 2))) * mmperdot * scale);**

**m_ptStart = new System.Drawing.Point(e.X, e.Y);**

**lp.Add(new System.Drawing.Point(e.X, e.Y));**

**}**

**}**

**}**

**else**

**{**

**if (!m_bMouseDown)**

**{**

**m_ptStart = new System.Drawing.Point(e.X, e.Y);**

**m_ptEnd = new System.Drawing.Point(e.X, e.Y);**

**m_bMouseDown = !m_bMouseDown;**

**}**

**else**

**{**

**m_ptStart = new System.Drawing.Point(e.X, e.Y);**

**}**

**}**

**if (imgpre.Image != null)**

**{**

**landscape = (imgpre.Width / imgpre.Height <= imgpre.Image.Width / imgpre.Image.Height);**

**if (landscape)**

**ratio = Convert.ToDouble(imgpre.Image.Width) / imgpre.Width;**

**else**

**ratio = Convert.ToDouble(imgpre.Image.Height) / imgpre.Height;**

**if (landscape)**

**{**

**imgleft = Convert.ToInt16(m_ptStart.X * ratio);**

**imgtop = Convert.ToInt16((m_ptStart.Y - (imgpre.Height - imgpre.Image.Height / ratio) / 2) * ratio);**

**}**

**else**

**{**

**imgleft = Convert.ToInt16((m_ptStart.X - (imgpre.Width - imgpre.Image.Width / ratio) / 2) * ratio);**

**imgtop = Convert.ToInt16(m_ptStart.Y * ratio);**

**}**

**double r = 0, g = 0, b = 0;**

**int n = 0**

**int i = 1;**

**int ctr_id = 0;**

**foreach (Contour<Point> ctr in contourList)**

**{**

**if (CvInvoke.cvPointPolygonTest(ctr.Ptr, new PointF(imgleft, imgtop), false) > 0)**

**{**

**ctr_id = i;**

**}**

**i++;**

**}**

**if (ctr_id == 0)**

**{ if (imageSource != null) imgpre.Image = imageSource.ToBitmap(); }**

**else showInfo(ctr_id, contourList[ctr_id - 1]);**

**}**

**locxy.Text = "X: " + imgleft + "; Y: " + imgtop + "; 倍率: " + scale.ToString("F2");**

**imgpre.Refresh();**

**}**

**}**

**private Contour<Point> getPart(Bitmap src) {**

**Contour<Point> res = null;**

**Image<Bgr, byte> IS = new Image<Bgr, byte>(src);**

**Image<Gray, Byte> imageGrayscale = IS.Convert<Gray, Byte>();**

**var imageThreshold = imageGrayscale.CopyBlank();**

**CvInvoke.cvThreshold(imageGrayscale, imageThreshold, 0, 255, Emgu.CV.CvEnum.THRESH.CV_THRESH_OTSU);**

**imageThreshold = imageThreshold.Not();**

**Contour<System.Drawing.Point> contours = imageThreshold.FindContours(CHAIN_APPROX_METHOD.CV_CHAIN_APPROX_SIMPLE, RETR_TYPE.CV_RETR_LIST);**

**while (contours != null)**

**{**

**res = contours;**

**break;**

**}**

**return res;**

**}**

**string[] fruitShape = new string[4];**

**string[] colorType = new string[5];**

**string[] colorType2 = new string[3];**

**string[] leafbase = new string[3];**

**string[] leaftop = new string[3];**

**string[] leafedge = new string[3];**

**private void showInfo(int i, Contour<Point> ctr)**

**{**

**double jz = 1;**

**if (cc.checkReg()) jz = 1-jz*DateTime.Now.Second / 120; double jzh = 1;**

**double mmperdot = 25.4 / Convert.ToDouble(imgdpi.Value);**

**rlist.Items[0].SubItems[1].Text = i.ToString();**

**if (fruitHeight.Value > 0)**

**{**

**jzh = 1 / (1 + (2.7013 * (double)fruitHeight.Value / 2 + 0.8335) / 100);**

**}**

**double para = mmperdot * scale * jz * jzh;**

**if (imgtype.SelectedIndex == 1)**

**{**

**MCvBox2D mar = ctr.GetMinAreaRect();**

**imageSource.Draw(mar, new Bgr(256, 0, 0), 1); float h = mar.size.Height;**

**float w = mar.size.Width;**

**int p = 1;**

**Rectangle rect = new Rectangle(ctr.BoundingRectangle.X - p, ctr.BoundingRectangle.Y - p, ctr.BoundingRectangle.Width + 2 * p, ctr.BoundingRectangle.Height + 2 * p);**

**Bitmap tmp = origin.Clone(mar.MinAreaRect(), System.Drawing.Imaging.PixelFormat.DontCare);**

**Seq<Point> convexHull = ctr.GetConvexHull(Emgu.CV.CvEnum.ORIENTATION.CV_CLOCKWISE);**

**Seq<MCvConvexityDefect> defects = ctr.GetConvexityDefacts(new MemStorage(), ORIENTATION.CV_CLOCKWISE);**

**MCvConvexityDefect bottomp = defects[0];**

**Point topp = convexHull[0];**

**int dfmax = bottomp.StartPoint.Y + bottomp.EndPoint.Y;**

**int dfmin = topp.Y;**

**for (int j = 0; j < defects.Total; j++)**

**{**

**if (defects[j].StartPoint.Y + defects[j].EndPoint.Y > dfmax && Math.Abs(defects[j].StartPoint.X - defects[j].EndPoint.X) > w / 10)**

**{**

**dfmax = defects[j].StartPoint.Y + defects[j].EndPoint.Y;**

**bottomp = defects[j];**

**}**

**}**

**for (int j = 0; j < convexHull.Total; j++)**

**{**

**if (convexHull[j].Y < dfmin)**

**{**

**dfmin = convexHull[j].Y;**

**topp = convexHull[j];**

**}**

**}**

**Point leftp = topp, rightp = topp;**

**foreach (Point pt in ctr)**

**{**

**double jl = Math.Sqrt(Math.Pow(topp.X - pt.X, 2) + Math.Pow(topp.Y - pt.Y, 2));**

**if (jl > w / 10 && jl < w / 7)**

**{**

**if (leftp == topp) { leftp = pt; }**

**else if (rightp == topp)**

**{**

**if (leftp.X - topp.X > 0 && pt.X - topp.X < 0) rightp = pt;**

**if (leftp.X - topp.X < 0 && pt.X - topp.X > 0) rightp = pt;**

**}**

**}**

**}**

**imageSource.Draw(new CircleF(bottomp.DepthPoint, 2), new Bgr(0, 256, 256), 2);**

**imageSource.Draw(new CircleF(bottomp.StartPoint, 2), new Bgr(256, 0, 256), 2);**

**imageSource.Draw(new CircleF(bottomp.EndPoint, 2), new Bgr(0, 256, 0), 2);**

**imageSource.Draw(new CircleF(topp, 2), new Bgr(0, 256, 256), 2);**

**imageSource.Draw(new CircleF(leftp, 2), new Bgr(0, 256, 0), 2);**

**imageSource.Draw(new CircleF(rightp, 2), new Bgr(256, 0, 256), 2);**

**rlist.Items[1].SubItems[1].Text = (h * para).ToString("F1");**

**rlist.Items[1].SubItems[1].Tag = (h * h).ToString();**

**rlist.Items[2].SubItems[1].Text = (w * para).ToString("F1");**

**rlist.Items[3].SubItems[1].Text = (ctr.Area* para*para / 100).ToString("F1");**

**rlist.Items[4].SubItems[1].Text = (w / h).ToString("F2");**

**rlist.Items[5].SubItems[1].Text = Angle(topp, leftp, rightp).ToString("F1");**

**rlist.Items[6].SubItems[1].Text = Angle(bottomp.DepthPoint, bottomp.StartPoint, bottomp.EndPoint).ToString("F1"); }**

**if (imgtype.SelectedIndex == 2)**

**{**

**imageSource.Draw(new LineSegment2D(pt1,pt2), new Bgr(256, 256, 0), 2);**

**thickness += Math.Sqrt(Math.Pow(pt1.X - pt2.X, 2) + Math.Pow(pt1.Y - pt2.Y, 2));**

**}**

**}**

**rlist.Items[7].SubItems[1].Text = (roomarea *para*para / 100).ToString("F1");**

**rlist.Items[8].SubItems[1].Text = rooms.ToString();**

**rlist.Items[9].SubItems[1].Text = (roomarea * para*para/ 100/rooms).ToString("F1");**

**rlist.Items[10].SubItems[1].Text = (roomarea*100/ctr.Area).ToString("F1");**

**rlist.Items[11].SubItems[1].Text = (thickness/rooms).ToString("F1");**

**}**

**}**

**if (imgtype.SelectedIndex == 0)**

**{**

**double r = 0, g = 0, b = 0;**

**int n = 0;**

**Rectangle mar2 = ctr.BoundingRectangle;**

**operating.Show();**

**Image<Hsv, byte> newimg2 = new Image<Hsv, byte>(imageSource.Size);**

**CvInvoke.cvCvtColor(imageSource, newimg2, COLOR_CONVERSION.BGR2HSV);**

**Image<Bgr, byte> newimg = imageSource.Copy();**

**for (int j = mar2.Left; j < mar2.Left + mar2.Width; j++) {**

**for (int k = mar2.Top; k < mar2.Top + mar2.Height; k++)**

**{**

**{**

**bool inhole = false;**

**if (excludehole.Checked)**

**{**

**foreach (Contour<Point> ctr2 in contourList)**

**{**

**if (ctr2.Area < average_area)**

**{**

**if (CvInvoke.cvPointPolygonTest(ctr2.Ptr, new PointF(j, k), false) > 0) {**

**inhole = true;**

**break;**

**}**

**}**

**}**

**}**

**if (!inhole)**

**{**

**MCvScalar s = CvInvoke.cvGet2D(newimg, k, j);**

**MCvScalar s2 = CvInvoke.cvGet2D(newimg2, k,j);**

**n++;**

**}**

**}**

**Application.DoEvents();**

**}**

**}**

**operating.Hide();**

**if (!clickstrips.Checked) imgpre.Image = newimg.ToBitmap();**

**rlist.Items[12].SubItems[1].Text = (r * jz / n).ToString("F1");**

**rlist.Items[13].SubItems[1].Text = (g * jz / n).ToString("F1");**

**rlist.Items[14].SubItems[1].Text = (b * jz / n).ToString("F1");**

**double LL = 0.2126007 * r + 0.7151947 * g + 0.0722046 * b;**

**double aa = 0.3258962 * r - 0.4992596 * g + 0.1733409 * b + 128;**

**double bb = 0.1218128 * r + 0.3785610 * g - 0.5003738 * b + 128;**

**rlist.Items[15].SubItems[1].Text = (LL * jz).ToString("F1");**

**rlist.Items[16].SubItems[1].Text = (aa * jz).ToString("F1"); rlist.Items[17].SubItems[1].Text = (bb * jz).ToString("F1");**

**rlist.Items[19].SubItems[1].Text = findColorName(r * jz / n, g * jz / n, b * jz / n);**

**rlist.Items[18].SubItems[1].Text =bweight.Text;**

**imgpre.Image = imageSource.ToBitmap();**

**}**

**string BaiduAccessToken = "";**

**string appid = ""; string appsecret = ""; string TokenUrl = "https:**

**public static String getFileBase64(String fileName)**

**{**

**FileStream filestream = new FileStream(fileName, FileMode.Open);**

**byte[] arr = new byte[filestream.Length];**

**filestream.Read(arr, 0, (int)filestream.Length);**

**string baser64 = Convert.ToBase64String(arr);**

**filestream.Close();**

**return baser64;**

**}**

**private void getrooms(string fn)**

**{**

**if (BaiduAccessToken == "") BaiduAccessToken = getAccessToken();**

**Console.WriteLine(BaiduAccessToken);**

**if (BaiduAccessToken != "")**

**{**

**if (File.Exists(fn))**

**{**

**operating.Text = "正在识别番茄心室，请稍后...";**

**operating.Show();**

**Application.DoEvents();**

**Encoding encoding = Encoding.Default;**

**HttpWebRequest request = (HttpWebRequest)WebRequest.Create(host);**

**request.Method = "post";**

**request.ContentType = "application/json";**

**request.KeepAlive = true;**

**String str = getFileBase64(fn);**

**str = "{\"image\":\"" + str + "\"}";**

**byte[] buffer = encoding.GetBytes(str);**

**request.ContentLength = buffer.Length;**

**request.GetRequestStream().Write(buffer, 0, buffer.Length);**

**HttpWebResponse response = (HttpWebResponse)request.GetResponse();**

**StreamReader reader = new StreamReader(response.GetResponseStream(), Encoding.UTF8);**

**string result = reader.ReadToEnd();**

**JavaScriptObject jsonObj = JavaScriptConvert.DeserializeObject<JavaScriptObject>(result);**

**if (jsonObj.ContainsKey("results"))**

**{**

**JavaScriptArray oo = jsonObj["results"] as JavaScriptArray;**

**oo.Count.ToString();**

**imgpre.Image = imageSource.ToBitmap();**

**origin = imageSource.ToBitmap();**

**operating.Hide();**

**operating.Text = "请右键打开或直接拖入多张JPG图片开始分析...";**

**Application.DoEvents();**

**}**

**else MessageBox.Show("未识别到番茄心室!");**

**}**

**}**

**}**

**private string getAccessToken()**

**{**

**string url = string.Format(TokenUrl, appid, appsecret);**

**string respText = "";**

**HttpWebRequest request = (HttpWebRequest)WebRequest.Create(url);**

**HttpWebResponse response = (HttpWebResponse)request.GetResponse();**

**using (Stream resStream = response.GetResponseStream())**

**{**

**StreamReader reader = new StreamReader(resStream, Encoding.UTF8);**

**respText = reader.ReadToEnd();**

**JavaScriptObject jsonObj = JavaScriptConvert.DeserializeObject<JavaScriptObject>(respText);**

**respText = jsonObj["access_token"].ToString();**

**resStream.Close();**

**}**

**return respText;**

**}**

**public static double Angle(Point cen, Point first, Point second)**

**{**

**const double M_PI = 3.1415926535897;**

**double ma_x = first.X - cen.X;**

**double ma_y = first.Y - cen.Y;**

**double mb_x = second.X - cen.X;**

**double mb_y = second.Y - cen.Y;**

**double v1 = (ma_x * mb_x) + (ma_y * mb_y);**

**double ma_val = Math.Sqrt(ma_x * ma_x + ma_y * ma_y);**

**double mb_val = Math.Sqrt(mb_x * mb_x + mb_y * mb_y);**

**double cosM = v1 / (ma_val * mb_val);**

**double angleAMB = Math.Acos(cosM) * 180 / M_PI;**

**return angleAMB;**

**}**

**public static PointF GetIntersection(Point lineFirstStar, Point lineFirstEnd, Point lineSecondStar, Point lineSecondEnd)**

**{**

**float a = 0, b = 0;**

**int state = 0;**

**if (lineFirstStar.X != lineFirstEnd.X)**

**{**

**a = (lineFirstEnd.Y - lineFirstStar.Y) / (lineFirstEnd.X - lineFirstStar.X);**

**state |= 1;**

**}**

**if (lineSecondStar.X != lineSecondEnd.X)**

**{**

**b = (lineSecondEnd.Y - lineSecondStar.Y) / (lineSecondEnd.X - lineSecondStar.X);**

**state |= 2;**

**}**

**switch (state)**

**{**

**case 0:**

**{**

**if (lineFirstStar.X == lineSecondStar.X)**

**{**

**return new PointF(0, 0);**

**}**

**else**

**{**

**return new PointF(0, 0);**

**}**

**}**

**case 1:**

**{**

**float x = lineSecondStar.X;**

**float y = (lineFirstStar.X - x) * (-a) + lineFirstStar.Y;**

**return new PointF(x, y);**

**}**

**case 2:**

**{**

**float x = lineFirstStar.X;**

**float y = (lineSecondStar.X - x) * (-b) + lineSecondStar.Y;**

**return new PointF(x, y);**

**}**

**case 3:**

**{**

**if (a == b)**

**{**

**return new PointF(0, 0);**

**}**

**float x = (a * lineFirstStar.X - b * lineSecondStar.X - lineFirstStar.Y + lineSecondStar.Y) / (a - b);**

**float y = a * x - a * lineFirstStar.X + lineFirstStar.Y;**

**return new PointF(x, y);**

**}**

**}**

**return new Point(0, 0);**

**}**

**private void imgcrop_Click(object sender, EventArgs e)**

**{**

**if (imgtop > 0 && imgH > 0 && imgleft > 0 && imgW > 0 & imgleft > 0 && imgW > 0)**

**{**

**Rectangle rect=new Rectangle(imgleft, imgtop, imgW, imgH);**

**origin = origin.Clone(rect, System.Drawing.Imaging.PixelFormat.DontCare);**

**imgpre.Image = origin;**

**checkFullScreen();**

**m_ptStart = m_ptEnd;**

**}**

**}**

**public static Bitmap Skelatanize(Image<Gray, byte> img2)**

**{**

**Image<Gray, byte> skel = new Image<Gray, byte>(img2.Size);**

**for (int y = 0; y < skel.Height; y++)**

**for (int x = 0; x < skel.Width; x++)**

**skel.Data[y, x, 0] = 0;**

**Image<Gray, byte> img = skel.Copy();**

**for (int y = 0; y < skel.Height; y++)**

**for (int x = 0; x < skel.Width; x++)**

**img.Data[y, x, 0] = img2.Data[y, x, 0];**

**StructuringElementEx element;**

**element = new StructuringElementEx(3, 3, 1, 1, Emgu.CV.CvEnum.CV_ELEMENT_SHAPE.CV_SHAPE_CROSS);**

**Image<Gray, byte> temp;**

**bool done = false;**

**do**

**{**

**temp = img.MorphologyEx(element, Emgu.CV.CvEnum.CV_MORPH_OP.CV_MOP_OPEN, 1);**

**temp = temp.Not();**

**temp = temp.And(img);**

**skel = skel.Or(temp);**

**img = img.Erode(1);**

**double[] min, max;**

**Point[] pmin, pmax;**

**img.MinMax(out min, out max, out pmin, out pmax);**

**done = (max[0] == 0);**

**} while (!done);**

**return skel.ToBitmap();**

**}**

**private void checkFullScreen()**

**{**

**inthumb.Checked = (imgpre.Image.Width > splitContainer1.Panel1.Width) || (imgpre.Image.Height> splitContainer1.Panel1.Height);**

**inthumb_Click(null, null);**

**}**

**List<Contour<Point>> contourList = new List<Contour<Point>>();**

**Image<Bgr, Byte> imageSource;**

**double scale = 1;**

**int maxWidth = 900;**

**double average_area = 0;**

**private void getres_Click(object sender, EventArgs e)**

**{**

**if (imgpre.Image != null && origin!=null)**

**{**

**imageSource = new Image<Bgr, byte>(origin);**

**Image<Gray, Byte> imageGrayscale = imageSource.Convert<Gray, Byte>();**

**if (grayway.SelectedIndex > 0) imageGrayscale = imageSource[grayway.SelectedIndex - 1];**

**double otsu_thresh_val = 0;**

**Image<Gray, Byte> imageThreshold = null;**

**if (binaryway.SelectedIndex == 0)**

**{**

**imageThreshold = imageGrayscale.CopyBlank();**

**otsu_thresh_val = CvInvoke.cvThreshold(imageGrayscale, imageThreshold, 0, 255, Emgu.CV.CvEnum.THRESH.CV_THRESH_OTSU);**

**}**

**else**

**{**

**imageThreshold = imageGrayscale.ThresholdBinary(new Gray(thr.Value),new Gray(255));**

**}**

**if (reduceNoise.Checked)**

**{**

**imageThreshold._Erode(1);**

**imageThreshold._Dilate(1);**

**}**

**Contour<System.Drawing.Point> contours = imageThreshold.FindContours(CHAIN_APPROX_METHOD.CV_CHAIN_APPROX_NONE,RETR_TYPE.CV_RETR_LIST);**

**int i = 1;**

**contourList.Clear();**

**while (contours != null)**

**{**

**if (contours.BoundingRectangle.Width > minwd.Value * origin.Width / basenum.Value && contours.BoundingRectangle.Width < maxwd.Value * origin.Width / basenum.Value**

**&& contours.BoundingRectangle.Height > minwd.Value * origin.Height / basenum.Value && contours.BoundingRectangle.Height < maxwd.Value * origin.Height / basenum.Value)**

**{**

**contourList.Add(contours);**

**}**

**contours = contours.HNext;**

**}**

**Contour<Point> temp = null;**

**for (int c = 0; c < contourList.Count; c++)**

**{**

**Rectangle rtc = contourList[c].BoundingRectangle;**

**int xy = rtc.X * 2 + rtc.Y * 10;**

**for (int cc = c + 1; cc < contourList.Count; cc++)**

**{**

**Rectangle rtc2 = contourList[cc].BoundingRectangle;**

**int jl = rtc2.X * 2 + rtc2.Y * 10;**

**if (jl < xy)**

**{**

**temp = contourList[cc];**

**contourList[cc] = contourList[c];**

**contourList[c] = temp;**

**xy = jl;**

**}**

**}**

**average_area += contourList[c].Area;**

**}**

**average_area = average_area / contourList.Count/2;**

**for (int c = 0; c < contourList.Count; c++)**

**{**

**if (contourList[c].Area < average_area)**

**{**

**for (int cc = c + 1; cc < contourList.Count; cc++)**

**{**

**if (contourList[cc].Area >= average_area) {**

**temp = contourList[cc];**

**contourList[cc] = contourList[c];**

**contourList[c] = temp;**

**break;**

**}**

**}**

**}**

**}**

**double otsu_thresh_low = otsu_thresh_val * 0.5;**

**if (binimage.Checked)**

**imgpre.Image = imageThreshold.ToBitmap();**

**else**

**imgpre.Image = imageSource.ToBitmap();**

**}**

**}**

**private void getebv_Click(object sender, EventArgs e)**

**{**

**getebv.Checked = !getebv.Checked;**

**if (getebv.Checked)**

**{**

**inthumb.Checked = false;**

**inthumb_Click(null, null);**

**}**

**}**

**private void saveres_Click(object sender, EventArgs e)**

**{**

**reslist.ListViewItemSorter = null;**

**int qs = reslist.Items.Count;**

**if (reslist.Columns.Count-1 != rlist.Items.Count)**

**{**

**reslist.Items.Clear();**

**reslist.Columns.Clear();**

**reslist.Columns.Add("图像编号");**

**for (int k = 0; k < rlist.Items.Count; k++) reslist.Columns.Add(rlist.Items[k].SubItems[0].Text);**

**}**

**int i = 1;**

**foreach (Contour<Point> ctr in contourList)**

**{**

**bool canread = true;**

**if (imgtype.SelectedIndex == 2) canread = (ctr.Area > 2*average_area);**

**if (canread)**

**{**

**showInfo(i, ctr);**

**ListViewItem lvi = reslist.Items.Add(imgid.Text);**

**for (int j = 0; j < rlist.Items.Count; j++)**

**{**

**lvi.SubItems.Add(rlist.Items[j].SubItems[1].Text);**

**}**

**}**

**i++;**

**Application.DoEvents();**

**}**

**ListViewItem lvi_avg = reslist.Items.Add(imgid.Text);**

**lvi_avg.SubItems.Add("平均值");**

**for (int j = 2; j < reslist.Columns.Count; j++)**

**{**

**double[] data = new double[contourList.Count];**

**for (int k = 0; k < contourList.Count; k++)**

**{**

**try**

**{**

**data[k] = Convert.ToDouble(reslist.Items[k+qs].SubItems[j].Text);**

**}**

**catch**

**{**

**data[k] = 0.0;**

**}**

**}**

**StaticTools tool = new StaticTools(data);**

**lvi_avg.SubItems.Add(tool.getAverage().ToString("F2"));**

**}**

**saveres.Text = "保存结果";**

**tabmain.SelectedIndex = 1;**

**}**

**private bool asc = true;**

**int selectedCol = 0;**

**private void reslist_ColumnClick(object sender, ColumnClickEventArgs e)**

**{**

**asc = !asc;**

**reslist.ListViewItemSorter = new ListViewItemComparer(e.Column, asc);**

**if (e.Column > 1)**

**{**

**selectedCol = e.Column;**

**double[] data;**

**if (reslist.SelectedItems.Count <= 1)**

**{**

**data = new double[reslist.Items.Count];**

**for (int i = 0; i < reslist.Items.Count; i++)**

**{**

**try**

**{**

**data[i] = Convert.ToDouble(reslist.Items[i].SubItems[e.Column].Text);**

**}**

**catch**

**{**

**data[i] = 0.0;**

**}**

**}**

**}**

**else**

**{**

**data = new double[reslist.SelectedItems.Count];**

**for (int i = 0; i < reslist.SelectedItems.Count; i++)**

**{**

**try**

**{**

**data[i] = Convert.ToDouble(reslist.SelectedItems[i].SubItems[e.Column].Text);**

**}**

**catch**

**{**

**data[i] = 0.0;**

**}**

**}**

**}**

**StaticTools tool = new StaticTools(data);**

**resStat.Text = reslist.Columns[e.Column].Text + "总数:" + data.Length + "\r\n\r\n";**

**resStat.Text += "平均值：" + tool.getAverage().ToString("F5") + "\r\n\r\n";**

**resStat.Text += "方差：" + tool.yangBenFangCha().ToString("F5") + "\r\n\r\n";**

**resStat.Text += "标准差：" + tool.yangBenBiaoZhunCha().ToString("F5") + "\r\n\r\n";**

**resStat.Text += "极差：" + tool.yangBenJiCha().ToString("F5") + "\r\n\r\n";**

**resStat.Text += "递增率：" + tool.yangBenDiZhengLv().ToString("F5") + "\r\n\r\n";**

**resStat.Text += "变异系数：" + tool.yangBenBianYiXiShu().ToString("F5") + "\r\n\r\n";**

**resStat.Text += "最小值：" + tool.getMin().ToString("F5") + "\r\n\r\n";**

**resStat.Text += "最大值：" + tool.getMax().ToString("F5") + "\r\n\r\n";**

**}**

**}**

**private void resDel_Click(object sender, EventArgs e)**

**{**

**if (reslist.SelectedItems.Count > 0)**

**{**

**if (MessageBox.Show("是否删除选择记录" + reslist.SelectedItems.Count.ToString() + "条?该操作不可恢复!", "删除", MessageBoxButtons.YesNo, MessageBoxIcon.Exclamation, MessageBoxDefaultButton.Button2) == DialogResult.Yes)**

**{**

**for (int i = 0; i < reslist.SelectedItems.Count; i++)**

**{**

**reslist.Items.Remove(reslist.SelectedItems[i]);**

**i--;**

**}**

**}**

**}**

**}**

**private void resclear_Click(object sender, EventArgs e)**

**{**

**if (MessageBox.Show("是否确认清空记录(含表头)条?该操作不可恢复!", "删除", MessageBoxButtons.YesNo, MessageBoxIcon.Exclamation, MessageBoxDefaultButton.Button2) == DialogResult.Yes)**

**{**

**reslist.Items.Clear();**

**reslist.Columns.Clear();**

**reslist.Columns.Add("图片编号");**

**}**

**}**

**private void imgsave_Click(object sender, EventArgs e)**

**{**

**savefd.Filter = "JPG文件|*.jpg";**

**savefd.FileName = imgid.Text;**

**if (savefd.ShowDialog() == DialogResult.OK)**

**{**

**imgpre.Image.Save(savefd.FileName, ImageFormat.Jpeg);**

**MessageBox.Show("图片已保存", "完成");**

**}**

**}**

**private void openjpg_Click(object sender, EventArgs e)**

**{**

**if (openfd.ShowDialog() == DialogResult.OK)**

**{**

**imgloadinit(openfd.FileName);**

**}**

**}**

**public bool isNUM(string s)**

**{**

**if (s == "")**

**{**

**return false;**

**}**

**else**

**{**

**Regex m_regex = new Regex("^(-?[0-9]*[.]*[0-9]{0,20})$");**

**return m_regex.IsMatch(s);**

**}**

**}**

**private void imgloadinit(string fname)**

**{**

**origin = new Bitmap(fname);**

**ExifManager em2 = new ExifManager(fname);**

**caminfo.Text = " ";**

**if (em2.Copyright != "") caminfo.Text += "光圈:" + em2.Copyright;**

**if (em2.Title != "") caminfo.Text += ",快门:" + em2.Title;**

**if (em2.Description != "") caminfo.Text += ",感光度:" + em2.Description;**

**if (isNUM(em2.Artist))**

**{**

**caminfo.Text += ",像素参照值:" + em2.Artist; imgdpi.Value = Convert.ToInt16(25.4f* origin.Width / (900 * 10*Convert.ToSingle(em2.Artist)));**

**}**

**em2.Dispose();**

**imageSource = new Image<Bgr, byte>(origin);**

**string[] fn = fname.ToLower().Replace(".jpg", "").Split('\\');**

**operating.Text = "计算中，请稍候...";**

**operating.Hide();**

**contourList.Clear();**

**scale = 1;**

**if (imageSource.Width > maxWidth)**

**{**

**scale = Convert.ToDouble(imageSource.Width) / maxWidth;**

**imageSource = imageSource.Resize(maxWidth, imageSource.Height * maxWidth / imageSource.Width, Emgu.CV.CvEnum.INTER.CV_INTER_NN);**

**origin = imageSource.ToBitmap();**

**}**

**imgpre.Image = origin;**

**checkFullScreen();**

**}**

**private Bitmap origin;**

**private void tabPage1_DragDrop(object sender, DragEventArgs e)**

**{**

**if (e.Data.GetDataPresent(DataFormats.FileDrop))**

**{**

**Array fa = ((System.Array)e.Data.GetData(DataFormats.FileDrop));**

**for (int i = 0; i < fa.Length; i++)**

**{**

**string fname = fa.GetValue(i).ToString();**

**if (fname.ToLower().EndsWith("jpg"))**

**{**

**imgloadinit(fname);**

**if(imgtype.SelectedIndex==2)**

**{**

**imageSource.Save(AppPath + "心室.jpg");**

**getrooms(AppPath + "心室.jpg");**

**}**

**if (hsvtreat.Checked)**

**{**

**treatHSV();**

**}**

**}**

**else MessageBox.Show("请打开JPG类型图片", "错误");**

**}**

**}**

**}**

**private void resExcel_Click(object sender, EventArgs e)**

**{**

**if (resExcel.Tag.ToString() != "0")**

**{**

**savefd.Filter = "Excel文件|*.xls";**

**if (savefd.ShowDialog() == DialogResult.OK)**

**{**

**string fn = savefd.FileName;**

**if (!fn.ToLower().EndsWith(".xls")) fn = fn + ".xls";**

**directOUT(fn, null);**

**}**

**}**

**else**

**{**

**string fn = resExcel.Tag.ToString();**

**if (File.Exists(fn))**

**{**

**try**

**{**

**string strConn;**

**strConn = "Provider=Microsoft.Jet.OLEDB.4.0;Data Source=" + fn + ";Extended Properties='Excel 8.0;HDR=False;IMEX=1'";**

**OleDbConnection OleConn = new OleDbConnection(strConn);**

**OleConn.Open();**

**String sql = "SELECT * FROM [Sheet1$]"; OleDbDataAdapter OleDaExcel = new OleDbDataAdapter(sql, OleConn);**

**DataSet OleDsExcel = new DataSet();**

**OleDaExcel.Fill(OleDsExcel, "Sheet1");**

**DataTable imdt = OleDsExcel.Tables[0];**

**if (imdt.Columns.Count == reslist.Columns.Count) {**

**for (int i = 0; i < reslist.Columns.Count; i++) imdt.Columns[i].ColumnName = reslist.Columns[i].Text;**

**for (int i = 0; i < reslist.Items.Count; i++) {**

**for (int j = 0; j < imdt.Rows.Count; j++)**

**{**

**if (imdt.Rows[j][0].ToString() == reslist.Items[i].Text && imdt.Rows[j][1].ToString() == reslist.Items[i].SubItems[1].Text)**

**{**

**imdt.Rows.RemoveAt(j);**

**j--;**

**}**

**}**

**}**

**for (int i = 0; i < reslist.Items.Count; i++)**

**{**

**imdt.Rows.Add(reslist.Items[i].Text);**

**for (int j = 1; j < reslist.Columns.Count; j++)**

**{**

**imdt.Rows[i][j] = reslist.Items[i].SubItems[j].Text;**

**}**

**}**

**OleConn.Close();**

**directOUT(fn, OleDsExcel);**

**}**

**else directOUT(fn, null);**

**}**

**catch (Exception err)**

**{**

**MessageBox.Show("导出数据失败,原因：" + err.Message, "错误",**

**MessageBoxButtons.OK, MessageBoxIcon.Information);**

**}**

**}**

**else directOUT(fn, null);**

**}**

**}**

**private void directOUT(string fn, DataSet dsxp)**

**{**

**if (dsxp == null)**

**{**

**dsxp = new DataSet();**

**DataTable table = new DataTable("res");**

**for (int i = 0; i < reslist.Columns.Count; i++)**

**table.Columns.Add(reslist.Columns[i].Text);**

**for (int i = 0; i < reslist.Items.Count; i++)**

**{**

**table.Rows.Add(reslist.Items[i].Text);**

**for (int j = 1; j < reslist.Columns.Count; j++)**

**{**

**table.Rows[i][j] = reslist.Items[i].SubItems[j].Text;**

**}**

**}**

**dsxp.Tables.Add(table);**

**}**

**WriteTable2Excel(dsxp, fn, "result", resExcel);**

**}**

**public bool WriteTable2Excel(DataSet ds, string fileName, string tp, ToolStripButton tsb)**

**{**

**if (ds.Tables.Count == 0 || fileName == string.Empty)**

**{**

**return false;**

**}**

**Microsoft.Office.Interop.Excel.Application excel = new Microsoft.Office.Interop.Excel.ApplicationClass();**

**int rowindex = 1;**

**int colindex = 0;**

**Microsoft.Office.Interop.Excel.Workbook work = excel.Workbooks.Add(true);**

**Application.DoEvents();**

**System.Data.DataTable table = ds.Tables[0];**

**foreach (DataColumn col in table.Columns)**

**{**

**colindex++;**

**excel.Cells[1, colindex] = col.ColumnName;**

**}**

**Application.DoEvents();**

**foreach (DataRow row in table.Rows)**

**{**

**rowindex++;**

**colindex = 0;**

**foreach (DataColumn col in table.Columns)**

**{**

**colindex++;**

**excel.Cells[rowindex, colindex] = row[col.ColumnName].ToString();**

**}**

**tsb.Text = (rowindex * 100 / table.Rows.Count).ToString() + "%";**

**Application.DoEvents();**

**}**

**excel.Visible = false;**

**excel.ActiveWorkbook.SaveAs(fileName, Microsoft.Office.Interop.Excel.XlFileFormat.xlExcel7, null, null, null, null, Microsoft.Office.Interop.Excel.XlSaveAsAccessMode.xlNoChange, null, null, null, null, null);**

**excel.Quit();**

**excel = null;**

**GC.Collect();**

**tsb.Text = "导 出";**

**MessageBox.Show("数据已导出:" + fileName, "完成");**

**return true;**

**}**

**private void showeb_Click(object sender, EventArgs e)**

**{**

**}**

**private void getbkv_Click(object sender, EventArgs e)**

**{**

**getbkv.Checked = !getbkv.Checked;**

**if (getbkv.Checked)**

**{**

**inthumb.Checked = false;**

**inthumb_Click(null, null);**

**}**

**}**

**private void copyrows_Click(object sender, EventArgs e)**

**{**

**if (reslist.SelectedItems.Count > 0)**

**{**

**string res = "";**

**for (int i = 0; i < reslist.SelectedItems.Count; i++)**

**{**

**res += reslist.SelectedItems[i].Text;**

**for (int j =1; j < reslist.Columns.Count; j++)**

**{**

**res += "\t" + reslist.SelectedItems[i].SubItems[j].Text;**

**}**

**res += "\r\n";**

**}**

**Clipboard.SetText(res);**

**}**

**}**

**private void softreg_Click(object sender, EventArgs e)**

**{**

**snreg.Show();**

**}**

**private void mcode_copy_Click(object sender, EventArgs e)**

**{**

**Clipboard.SetText(mcode.Text);**

**}**

**common cc = new common();**

**private string findColorName(double r, double g, double b) {**

**double jl = 255 * 3;**

**int k = 0;**

**int j = 0;**

**string[] cl;**

**foreach (string s in rgb)**

**{**

**cl = s.Split('$');**

**if (cl.Length >= 6)**

**{**

**Double i = Math.Abs(r - Convert.ToDouble(cl[3])) + Math.Abs(g - Convert.ToDouble(cl[4])) + Math.Abs(b - Convert.ToDouble(cl[5]));**

**if (i < jl)**

**{**

**jl = i;**

**k = j;**

**}**

**}**

**j++;**

**}**

**cl = rgb[k].Split('$');**

**return cl[1];**

**}**

**private void Form1_FormClosed(object sender, FormClosedEventArgs e)**

**{**

**cc.setKeyValue("DPI_FruitHelper", imgdpi.Value.ToString());**

**cc.setKeyValue("minwidth_FruitHelper", minwd.Value.ToString());**

**cc.setKeyValue("maxwidth_FruitHelper", maxwd.Value.ToString());**

**cc.setKeyValue("fruitHeight_FruitHelper", fruitHeight.Value.ToString());**

**cc.setKeyValue("thrValue_FruitHelper", thr.Value.ToString());**

**cc.setKeyValue("maxhu_FruitHelper", maxhu.Value.ToString());**

**cc.setKeyValue("minhu_FruitHelper", minhu.Value.ToString());**

**cc.setKeyValue("vshift_FruitHelper", vshift.Value.ToString());**

**cc.setKeyValue("sshift_FruitHelper", sshift.Value.ToString());**

**if (_capture != null)**

**{**

**_capture.Stop();**

**_capture.ImageGrabbed -= ProcessFrame;**

**_capture.Dispose();**

**}**

**CameraHandler.Dispose();**

**}**

**private void setTag(Control cons)**

**{**

**foreach (Control con in cons.Controls)**

**{**

**con.Tag = con.Width + ":" + con.Height + ":" + con.Left + ":" + con.Top + ":" + con.Font.Size;**

**if (con is ToolStrip)**

**{**

**foreach (ToolStripItem tsmi in (con as ToolStrip).Items)**

**{**

**tsmi.Tag = tsmi.Width + ":" + tsmi.Height + ":0:0:" + tsmi.Font.Size;**

**}**

**}**

**if (con.Controls.Count > 0)**

**setTag(con);**

**}**

**}**

**private void setControls(float newx, float newy, Control cons)**

**{**

**foreach (Control con in cons.Controls)**

**{**

**if (con != resStat)**

**{**

**string[] mytag = con.Tag.ToString().Split(new char[] { ':' });**

**float a = Convert.ToSingle(mytag[0]) * newx;**

**con.Width = (int)a;**

**a = Convert.ToSingle(mytag[1]) * newy;**

**con.Height = (int)(a);**

**a = Convert.ToSingle(mytag[2]) * newx;**

**con.Left = (int)(a);**

**a = Convert.ToSingle(mytag[3]) * newy;**

**con.Top = (int)(a);**

**Single currentSize = Convert.ToSingle(mytag[4]) * newy;**

**con.Font = new Font(con.Font.Name, currentSize, con.Font.Style, con.Font.Unit);**

**if (con.Controls.Count > 0)**

**{**

**setControls(newx, newy, con);**

**}**

**if (con is ToolStrip)**

**{**

**foreach (ToolStripItem tsmi in (con as ToolStrip).Items)**

**{**

**mytag = tsmi.Tag.ToString().Split(new char[] { ':' });**

**currentSize = Convert.ToSingle(mytag[4]) * newy;**

**tsmi.Font = new Font(tsmi.Font.Name, currentSize, tsmi.Font.Style, tsmi.Font.Unit);**

**}**

**}**

**}**

**}**

**}**

**private void scanImg_Click(object sender, EventArgs e)**

**{**

**try**

**{**

**if (!gp_camera.Visible)**

**{**

**operating.Hide();**

**gp_camera.Show();**

**if (_capture != null)**

**{**

**_capture.Stop();**

**_capture.ImageGrabbed -= ProcessFrame;**

**_capture = null;**

**}**

**if (_capture == null)**

**{**

**_capture = new Emgu.CV.Capture(Camera_Selection.SelectedIndex);**

**string[] r = resolutions.Text.Split('*');**

**if (r[0].Length> 0 && r[1].Length>0)**

**{**

**_capture.SetCaptureProperty(Emgu.CV.CvEnum.CAP_PROP.CV_CAP_PROP_FRAME_WIDTH, Convert.ToInt16(r[0]));**

**_capture.SetCaptureProperty(Emgu.CV.CvEnum.CAP_PROP.CV_CAP_PROP_FRAME_HEIGHT, Convert.ToInt16(r[1]));**

**}**

**_capture.ImageGrabbed += ProcessFrame;**

**cam_snap.Enabled = true;**

**}**

**_capture.Start();**

**Application.DoEvents();**

**}**

**}**

**catch { MessageBox.Show("请检查高拍仪是否连接正常!", "错误");}**

**}**

**private void basenum_ValueChanged(object sender, EventArgs e)**

**{**

**maxwd.Maximum = basenum.Value;**

**}**

**private void functionp_DoubleClick(object sender, EventArgs e)**

**{**

**float newy = this.Height / Y;**

**functionp.Height = Convert.ToInt16(370 * newy);**

**}**

**private void copycol_Click(object sender, EventArgs e)**

**{**

**if (selectedCol > 1)**

**{**

**string res = "";**

**for (int i = 0; i < reslist.Items.Count; i++)**

**{**

**res += reslist.Items[i].Text;**

**res += "\t" + reslist.Items[i].SubItems[1].Text;**

**res += "\t" + reslist.Items[i].SubItems[selectedCol].Text;**

**}**

**res += "\r\n";**

**}**

**Clipboard.SetText(res);**

**}**

**}**

**private void rotate90_Click(object sender, EventArgs e)**

**{**

**if (origin != null)**

**{**

**imageSource = new Image<Bgr, byte>(origin);**

**imageSource = imageSource.Rotate(90, new Bgr(0, 0, 0),false);**

**origin = imageSource.ToBitmap();**

**imgpre.Image = origin;**

**}**

**}**

**private void thr_ValueChanged(object sender, EventArgs e)**

**{**

**thr_l.Text = "黑<" + thr.Value.ToString();**

**thr_r.Text = "白>" + thr.Value.ToString();**

**getres_Click(sender, e);**

**}**

**private void binaryway_SelectedIndexChanged(object sender, EventArgs e)**

**{**

**thr.Enabled = (binaryway.SelectedIndex == 1);**

**getres_Click(sender, e);**

**}**

**private void getresolution_Click(object sender, EventArgs e)**

**{**

**if (rlist.Items[1].SubItems[1].Tag.ToString() != "")**

**{**

**double n = Convert.ToDouble(rlist.Items[1].SubItems[1].Tag);**

**decimal dpi = Convert.ToDecimal(25.4f / Math.Sqrt(100f / (n * scale * scale)));**

**if (dpi > imgdpi.Maximum)**

**MessageBox.Show("无效参照物!", "错误");**

**else imgdpi.Value = dpi;**

**}**

**}**

**private void cam_close_Click(object sender, EventArgs e)**

**{**

**gp_camera.Hide();**

**operating.Show();**

**_capture.Stop();**

**imgpre.Image = null;**

**imgpre.Refresh();**

**}**

**private void Camera_Selection_SelectedIndexChanged(object sender, EventArgs e)**

**{**

**resolutions.Items.Clear();**

**int r = 0;**

**foreach (string s in GetAllAvailableResolution(_SystemCamereas[Camera_Selection.SelectedIndex]))**

**{**

**if (!resolutions.Items.Contains(s))**

**{**

**resolutions.Items.Add(s);**

**if (s.StartsWith("1024")) resolutions.SelectedIndex = r;**

**r++;**

**}**

**}**

**}**

**private List<string> GetAllAvailableResolution(DsDevice vidDev)**

**{**

**try**

**{**

**int hr, bitCount = 0;**

**IBaseFilter sourceFilter = null;**

**var m_FilterGraph2 = new FilterGraph() as IFilterGraph2;**

**hr = m_FilterGraph2.AddSourceFilterForMoniker(vidDev.Mon, null, vidDev.Name, out sourceFilter);**

**var pRaw2 = DsFindPin.ByCategory(sourceFilter, PinCategory.Capture, 0);**

**var AvailableResolutions = new List<string>();**

**VideoInfoHeader v = new VideoInfoHeader();**

**IEnumMediaTypes mediaTypeEnum;**

**hr = pRaw2.EnumMediaTypes(out mediaTypeEnum);**

**AMMediaType[] mediaTypes = new AMMediaType[1];**

**IntPtr fetched = IntPtr.Zero;**

**hr = mediaTypeEnum.Next(1, mediaTypes, fetched);**

**while (fetched != null && mediaTypes[0] != null)**

**{**

**Marshal.PtrToStructure(mediaTypes[0].formatPtr, v);**

**if (v.BmiHeader.Size != 0 && v.BmiHeader.BitCount != 0)**

**{**

**if (v.BmiHeader.BitCount > bitCount)**

**{**

**AvailableResolutions.Clear();**

**bitCount = v.BmiHeader.BitCount;**

**}**

**AvailableResolutions.Add(v.BmiHeader.Width + "*" + v.BmiHeader.Height);**

**}**

**hr = mediaTypeEnum.Next(1, mediaTypes, fetched);**

**}**

**return AvailableResolutions;**

**}**

**catch (Exception ex)**

**{**

**return new List<string>();**

**}**

**}**

**private void cam_snap_Click(object sender, EventArgs e)**

**{**

**if (imgpre.Image != null)**

**{**

**gp_camera.Hide();**

**_capture.Stop();**

**origin = new Bitmap(imgpre.Image);**

**imgpre.Refresh();**

**operating.Hide();**

**imgid.Text = DateTime.Now.ToString("yyyyMMdd-hhmmss");**

**}**

**}**

**private void imgrotate_SelectedIndexChanged(object sender, EventArgs e)**

**{**

**if (_capture != null)**

**{**

**if (imgrotate.SelectedIndex == 1)**

**_capture.FlipHorizontal = !_capture.FlipHorizontal;**

**else if (imgrotate.SelectedIndex == 2)**

**_capture.FlipVertical = !_capture.FlipVertical;**

**}**

**public static void ImgTranslate(Image<Bgr, Byte> srcImg, Image<Bgr, Byte> dstImg, int xOffset, int yOffset)**

**{**

**for (int i = 0; i < srcImg.Rows; i++)**

**{**

**for (int j = 0; j < srcImg.Cols; j++)**

**{**

**int x = j + xOffset;**

**int y = i + yOffset;**

**if (x >= 0 && x < dstImg.Cols && y >= 0 && y < dstImg.Rows)**

**dstImg[y, x] = srcImg[i, j];**

**}**

**}**

**}**

**private void button2_Click(object sender, EventArgs e)**

**{**

**string strFileName = string.Empty;**

**OpenFileDialog ofd = new OpenFileDialog();**

**if (ofd.ShowDialog() == DialogResult.OK)**

**{**

**Image<Bgr, Byte> img_2 = new Image<Bgr, Byte>(ofd.FileName); img2 = img_2.Convert<Gray, Byte>();**

**pictureBox2.Image = img2.ToBitmap();**

**}**

**}**

**private void hsvtreat_Click(object sender, EventArgs e)**

**{**

**hsvpanel.Visible = hsvtreat.Checked;**

**if(hsvtreat.Checked)**

**{**

**treatHSV();**

**}**

**}**

**private void leafcolor_SelectedValueChanged(object sender, EventArgs e)**

**{**

**switch (leafcolor.SelectedIndex)**

**{**

**case 0:**

**minhu.Value = 26;**

**maxhu.Value = 77;**

**break;**

**case 1:**

**minhu.Value = 35;**

**maxhu.Value = 77;**

**break;**

**case 2:**

**minhu.Value = 26;**

**maxhu.Value = 34;**

**break;**

**case 3:**

**minhu.Value = 125;**

**maxhu.Value = 155;**

**break;**

**case 4:**

**minhu.Value = 156;**

**maxhu.Value = 180;**

**break;**

**}**

**}**

**private void imgtype_SelectedIndexChanged(object sender, EventArgs e)**

**{**

**foreach (ListViewItem lvi in rlist.Items)**

**{**

**lvi.SubItems[1].Text = "";**

**}**

**}**

**private void drawwhiteline_CheckedChanged(object sender, EventArgs e)**

**{**

**if (drawwhiteline.Checked)**

**{**

**autolength.Checked = false;**

**imgpre.Dock = DockStyle.None;**

**imgpre.SizeMode = PictureBoxSizeMode.AutoSize;**

**m_ptStart = m_ptEnd;**

**lp.Clear();**

**}**

**else**

**{**

**this.Cursor = Cursors.Default;**

**}**

**imgpre.Refresh();**

**}**

**private void drawwhiteline_Click(object sender, EventArgs e)**

**{**

**}**

**private void drawwhiteline_Click_1(object sender, EventArgs e)**

**{**

**}**

**private void button3_Click(object sender, EventArgs e)**

**{**

**result = img1.AbsDiff(img2);**

**pictureBox3.Image = result.ToBitmap();**

**}**

**private void canonconfig_Click(object sender, EventArgs e)**

**{**

**if(canonpanel.Height<300)**

**canonpanel.Height = 510;**

**else**

**canonpanel.Height = 50;**

**canonpanel.Tag = canonpanel.Width + ":" + canonpanel.Height + ":" + canonpanel.Left + ":" + canonpanel.Top + ":" + canonpanel.Font.Size;**

**}**

**private void canonphoto_Click(object sender, EventArgs e)**

**{**

**try**

**{**

**if (!gp_camera.Visible)**

**{**

**operating.Hide();**

**RefreshCamera();**

**canonpanel.Show();**

**Application.DoEvents();**

**}**

**}**

**catch { MessageBox.Show("请检查相机是否连接正常!", "错误"); }**

**}**

**double changd = 0;**

**List<System.Drawing.Point> lp = new List<System.Drawing.Point>();**

**private void autolength_CheckedChanged(object sender, EventArgs e)**

**{**

**if (autolength.Checked)**

**{**

**drawwhiteline.Checked = false;**

**imgpre.Dock = DockStyle.None;**

**imgpre.SizeMode = PictureBoxSizeMode.AutoSize;**

**m_ptStart = m_ptEnd;**

**lp.Clear();**

**}**

**else**

**{**

**this.Cursor = Cursors.Default;**

**}**

**imgpre.Refresh();**

**}**

**private void imgpre_MouseDoubleClick(object sender, MouseEventArgs e)**

**{**

**if (lp.Count > 1)**

**{**

**if (drawwhiteline.Checked)**

**{**

**imageSource.DrawPolyline(lp.ToArray(), false, new Bgr(255, 255, 255), 2);**

**origin = new Bitmap(imageSource.ToBitmap());**

**imgpre.Image = origin;**

**}**

**else**

**{**

**MessageBox.Show("长度为" + changd.ToString("F2") + "mm,该数值已复制!", "测长");**

**Clipboard.SetText(changd.ToString("F2"));**

**}**

**}**

**m_bMouseDown = !m_bMouseDown;**

**imgpre.Refresh();**

**}**

**private void FocusFar2Button_Click(object sender, EventArgs e)**

**{**

**CameraHandler.SetFocus(EDSDK.EvfDriveLens_Far2);**

**}**

**private void FocusNear2Button_Click(object sender, EventArgs e)**

**{**

**CameraHandler.SetFocus(EDSDK.EvfDriveLens_Near2);**

**}**

**}**

**}**

**class ListViewItemComparer : IComparer**

**{**

**private int col;**

**private bool sorts;**

**public ListViewItemComparer()**

**{**

**col = 0;**

**sorts = true;**

**}**

**public ListViewItemComparer(int column, bool sort)**

**{**

**col = column;**

**sorts = sort;**

**}**

**public int Compare(object x, object y)**

**{**

**try**

**{**

**string xv = ((ListViewItem)x).SubItems[col].Text;**

**string yv = ((ListViewItem)y).SubItems[col].Text;**

**if (xv.Contains("平均值")) xv = "99999";**

**if (yv.Contains("平均值")) yv = "99999";**

**double xx = Convert.ToDouble(xv);**

**double yy = Convert.ToDouble(yv);**

**double res = 0;**

**if (sorts)**

**res = xx - yy;**

**else**

**res = yy - xx;**

**if (res == 0) return 0;**

**if (res > 0)**

**return 1;**

**else**

**return -1;**

**}**

**catch**

**{**

**if (sorts) return String.CompareOrdinal(((ListViewItem)x).SubItems[col].Text, ((ListViewItem)y).SubItems[col].Text);**

**else return String.CompareOrdinal(((ListViewItem)y).SubItems[col].Text, ((ListViewItem)x).SubItems[col].Text);**

**}**

**}**

**}**

**}**
