# Supplementary material for: Quantitative Extraction and Evaluation of Tomato Fruit Phenotypes Based on Image Recognition
Source: Front Plant Sci. 2022 Apr 13;13:859290. doi: 10.3389/fpls.2022.859290 (PMC9044966; doi:10.3389/fpls.2022.859290)
Supplement: Supplementary file 2 [file Data_Sheet_2.docx]

Supplementary Material

**Table S1 Manual and image-based phenotyping results of tomato fruits**

| Cultivar No. | Fruit color R mean | Fruit color G mean | Fruit color B mean | Horizontal diameter / mm | |  | Vertical diameter / mm | | Fruit top angle / ° | Fruit navel angle / ° | Pulp thickness / mm | |  | Locule number | | Locule area proportion / % |
| --- | --- | --- | --- | --- | --- | --- | --- | --- | --- | --- | --- | --- | --- | --- | --- | --- |
|  |  |  |  | Manual ^a^ | Recognition |  | Manual | Recognition |  |  | Manual | Recognition |  | Manual | Recognition |  |
| No. 405 | 146.33 | 138.00 | 56.03 | 75.80 ± 1.30 | 77.21 ± 1.62 |  | 63.10 ± 1.67 | 65.30 ± 1.26 | 158.58 ± 2.77 | 142.23 ± 3.58 | 9.76 ± 0.52 | 9.97 ± 0.24 |  | 4.250 | 4.250 | 25.63 ± 2.58 |
| No. 459 | 172.93 | 99.58 | 22.03 | 34.83 ± 1.51 | 35.94 ± 1.29 |  | 33.05 ± 2.09 | 33.62 ± 1.43 | 161.05 ± 5.95 | 149.25 ± 5.35 | 5.27 ± 0.54 | 5.15 ± 0.34 |  | 1.500 | 1.250 | 39.50 ± 3.32 |
| No. 106 | 177.43 | 92.93 | 11.60 | 72.47 ± 4.26 | 71.69 ± 2.17 |  | 62.23 ± 2.09 | 62.93 ± 1.69 | 164.93 ± 2.51 | 149.60 ± 3.44 | 8.67 ± 0.37 | 8.65 ± 0.29 |  | 3.500 | 3.500 | 28.00 ± 2.14 |
| No. 68 | 149.55 | 72.98 | 12.50 | 60.48 ± 1.02 | 61.88 ± 1.76 |  | 54.20 ± 3.64 | 54.85 ± 1.32 | 156.45 ± 2.41 | 142.13 ± 4.84 | 6.36 ± 0.95 | 6.32 ± 0.58 |  | 2.500 | 2.500 | 21.13 ± 1.68 |
| No. 129 | 168.30 | 71.58 | 18.03 | 47.80 ± 1.04 | 49.16 ± 1.32 |  | 41.65 ± 1.40 | 41.32 ± 1.10 | 161.55 ± 6.29 | 145.80 ± 4.41 | 6.28 ± 0.86 | 6.32 ± 0.31 |  | 3.000 | 3.000 | 34.98 ± 3.09 |
| No. 522 | 153.38 | 49.93 | 18.55 | 47.75 ± 1.03 | 47.60 ± 1.41 |  | 52.73 ± 2.29 | 53.94 ± 1.13 | 135.45 ± 4.78 | 152.38 ± 1.99 | 6.06 ± 0.31 | 5.83 ± 0.10 |  | 2.500 | 2.500 | 27.25 ± 1.56 |
| No. 80 | 154.93 | 37.80 | 20.78 | 92.93 ± 6.18 | 93.12 ± 2.11 |  | 66.02 ± 4.44 | 65.37 ± 2.04 | 166.68 ± 6.35 | 145.43 ± 5.62 | 10.31 ± 0.92 | 10.03 ± 0.54 |  | 3.125 | 3.125 | 30.05 ± 1.13 |
| No. 341 | 130.18 | 35.73 | 26.78 | 25.37 ± 1.33 | 25.11 ± 1.21 |  | 33.72 ± 1.58 | 33.69 ± 1.12 | 152.78 ± 4.02 | 155.38 ± 2.35 | 4.67 ± 0.24 | 4.81 ± 0.18 |  | 2.000 | 2.000 | 26.50 ± 2.40 |
| No. 123 | 82.93 | 48.83 | 29.73 | 33.27 ± 1.60 | 33.54 ± 1.58 |  | 36.03 ± 2.06 | 36.68 ± 1.34 | 159.78 ± 3.05 | 162.40 ± 2.31 | 3.90 ± 0.17 | 3.85 ± 0.09 |  | 2.500 | 2.500 | 29.29 ± 3.01 |
| No. 113 | 71.35 | 42.65 | 22.90 | 57.05 ± 1.45 | 56.93 ± 1.14 |  | 42.75 ± 1.54 | 43.13 ± 1.49 | 164.28 ± 6.90 | 149.43 ± 2.32 | 4.68 ± 0.49 | 4.86 ± 0.32 |  | 4.500 | 4.500 | 28.68 ± 0.95 |

^a^ **Manual** refers to manual measurement; **Recognition** refers to results of the image recognition method or the deep learning model.





**Figure S1 |** Training and validation of the deep learning segmentation model. **(A)** Training and validation loss curve; **(B)** F1-score over different thresholds.
